# Bronze Age make-up recipes from Sudanese Lower Nubia point to a greater diversity across cultural borders in ancient Northeast Africa

**DOI:** 10.1371/journal.pone.0330205

**Published:** 2025-09-11

**Authors:** Rennan Lemos, Caterina Zaggia, Kate Fulcher, Einar Lidén, Ludmila Werkström, Emma Hocker, Jonas Bergquist, Marcos Martinón-Torres

**Affiliations:** 1 Department of Archaeology, Durham University, Durham, United Kingdom; 2 The Bartlett School of Environment, Energy and Resources, University College London, London, United Kingdom; 3 Institute of Archaeology, University College London, London, United Kingdom; 4 Department of Chemistry, Uppsala University, Uppsala, Sweden; 5 Gustavianum, Uppsala University Museum, Uppsala, Sweden; 6 Department of Archaeology, University of Cambridge, Cambridge, United Kingdom; Loyola University Chicago, UNITED STATES OF AMERICA

## Abstract

Previous scientific explorations of kohl and other make-up substances from ancient Egypt have revealed a considerable diversity of materials and recipes used in different regions and time periods. However, samples from Sudanese Nubia have never been included in scientific investigations of make-up substances used along the Nile valley. For the first time, 24 samples of kohl and other cosmetics from Bronze Age Sudanese Lower Nubia (c. 2055–1070 BCE) were analysed using optical microscopy, GC-MS, SEM-EDS, ATR-FTIR and XRD. Beyond expanding our knowledge of make-up usage in the ancient Nile valley by including samples from Sudan, this study adds further depth to our understanding of make-up substances in ancient Northeast Africa by exploring samples from well-defined archaeological contexts. The multi-analytical approach presented here sheds light on the diversity of recipes used by various communities in the Middle Nile valley during the Bronze Age. Most samples are dominated by lead sulphides, but these occur in various mixtures with quartz, clay, calcite, gypsum and zinc compounds, in addition to plant gums and animal fats. We also report for the first time the use of synthetic calcium antimonate in ancient cosmetic mixtures. Besides expanding our knowledge of make-up mixtures in ancient Northeast Africa, our study suggests that the considerable variation detected across the cultural borders of Bronze Age Egypt and Nubia reflects distinctive bodily ideals.

## Introduction

Cosmetic products and associated material culture have been found in ancient Nile valley graves from the late Neolithic into dynastic times [1]. Among the cosmetic sets used in the region, kohl was widely popular from the Early Bronze Age [2]. Make-up containers from ancient Egypt are mostly found in burial contexts, from simple pit graves, like the ones discussed here, to monumental rock-cut tombs [3]. Amid diverse make-up recipes, the use of kohl is mentioned in ancient Egyptian love poems [4]. Make-up containers reached C-Group communities in Lower Nubia (c. 2300–1600 BCE) and Kerma people in Upper Nubia (c. 2050–1500 BCE) through trade and exchanges with Egypt [5–7]. The Egyptian occupation of Lower Nubia in the Middle Kingdom (c. 1975–1773 BCE), and later colonisation of both Lower and Upper Nubia in the New Kingdom (c. 1550–1070 BCE) made Egyptian-style make-up containers more widespread in Nubia [8,9].

Egyptian kohl has been extensively analysed [10]. Most of the analysed samples were tested for inorganic components and consist of a lead-based substance [11–17]. The usual black mixture was achieved by crushing galena and mixing it with other substances to achieve a usable product that could be applied to the body, especially around the eyes. Kohl substances are found in graves both in the form of a dry paste shaped after the container in which it was used, or as fine powder. For a long time, this has caused some confusion regarding kohl mixtures [18], especially as the vast majority of compositional studies of ancient Egyptian kohl have focused on inorganic analyses. However, recent analyses of kohl samples have detected the use of both plant and animal fatty materials, which were mixed with mineral components to achieve the final product [19]. Despite the predominance of lead-based mixtures in the analysed data sets, recent analyses have also suggested that a greater compositional diversity characterised ancient Egyptian make-up recipes, including silica-, manganese- and carbon-based substances [19]. The present study expands our knowledge of this diversity by unveiling further, perhaps culturally-specific recipes. At the same time, the study suggests that a less restrictive terminology should be adopted to account for a more diverse range of recipes used alongside kohl-mixtures.

**Fig 1:**
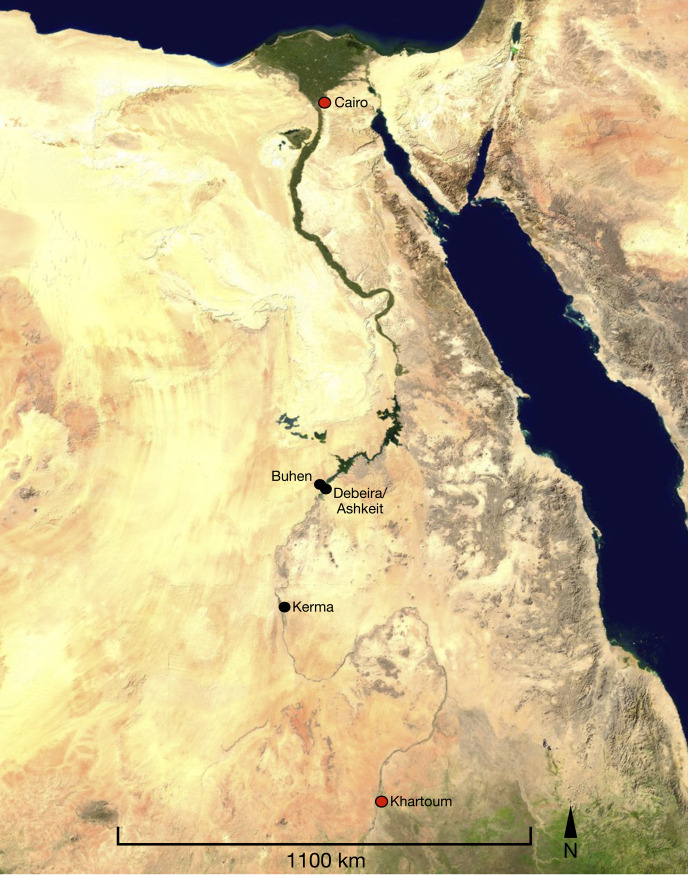
Map of Egypt and Nubia showing the sites mentioned in the text. Prepared by R. Lemos based on Wikimedia Commons image.

**Fig 2:**
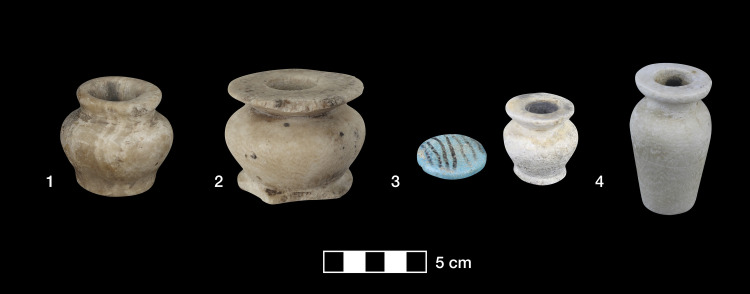
Make-up containers from (1) Buhen (sample 20), (2) Ashkeit/Site 183 (sample 15) and (3, 4) Debeira/Fadrus (samples 3 and 5, respectively). Photos by R. Lemos, courtesy of Gustavianum, Uppsala University Museum and the Museum of Archaeology and Anthropology, University of Cambridge.

This study significantly expands the scope of previous analyses of make-up substances from ancient Northeast Africa carried out since the 1930s by including, for the first time, a large data set of Lower Nubian samples from North Sudan dating from the Middle and Late Bronze Age (Fig 1). Samples taken from make-up containers from Debeira East, Ashkeit and Buhen kept at Gustavianum, Uppsala University Museum, and the Museum of Archaeology and Anthropology, University of Cambridge, were analysed for both organic and inorganic components (Fig 2). The results reveal that the majority of samples tested were lead-based mixtures, but a few samples add further elements of a greater diversity to our knowledge of ancient Nile valley cosmetics, as suggested by the most recently published results. In contrast with previous studies of poorly provenanced kohl, this study explores 22 securely dated and well-provenanced samples coming mostly from intact graves [20,21]. Two further samples were taken from containers found in properly defined archaeological deposits in a temple context [22]. These contribute to expanding our knowledge of ancient Nile valley cosmetic substances also by including samples from non-burial contexts (Table 1).

**Table 1:** Summary and contextualisation of all analysed samples. Archaeological data based on [20,21] and [22]. Samples 19 and 20 were taken from containers kept at the Museum of Archaeology and Anthropology, University of Cambridge, while the remaining samples come from containers kept at Gustavianum, Uppsala University Museum.

Sample	Site	Burial:Object	Date	Context	Techniques used
1	Debeira East/Site 185 (Fadrus)	200:7	New Kingdom	Side niche burial containing faience beads, scarabs and pottery	OM, SEM-EDS, ATR-FTIR, GC-MS
2	Debeira East/Site 185 (Fadrus)	2:1	New Kingdom	Pit burial containing a spiral gold earring	OM, SEM-EDS, ATR-FTIR, GC-MS
3	Debeira East/Site 185 (Fadrus)	22:1	New Kingdom	Pit burial containing 4 bronze spiral earrings	OM, SEM-EDS, ATR-FTIR, GC-MS
4	Debeira East/Site 185 (Fadrus)	56:2	New Kingdom	Pit burial (adult) containing a coffin, a scarab and pottery	OM, SEM-EDS, ATR-FTIR, GC-MS
5	Debeira East/Site 185 (Fadrus)	73:2	New Kingdom	Pit burial (adult female) containing 2 other kohl containers and pottery	OM, SEM-EDS, ATR-FTIR, GC-MS
6	Debeira East/Site 185 (Fadrus)	84:33	New Kingdom	Side niche burial (one adult and one infant) containing a mask with traces of gold foil, gold penannular earrings and several pottery vessels, including one Kerma beaker	OM, SEM-EDS, ATR-FTIR, GC-MS
7	Debeira East/Site 185 (Fadrus)	128:2 (lip)	New Kingdom	Pit burial containing a scarab, a pair of bronze tweezers and pottery	OM, SEM-EDS, GC-MS
8	Debeira East/Site 185 (Fadrus)	128:2 (bottom of container)	New Kingdom	See above	OM, SEM-EDS, ATR-FTIR, GC-MS
9	Debeira East/Site 185 (Fadrus)	246:10	New Kingdom	Mud-brick chamber in pit containing ivory inlay fragments and pottery	OM, SEM-EDS, ATR-FTIR, GC-MS
10	Debeira East/Site 185 (Fadrus)	248:7	New Kingdom	Side niche burial containing a cartonnage coffin, a scarab and pottery	OM, SEM-EDS, ATR-FTIR, GC-MS
11	Debeira East/Site 185 (Fadrus)	269:1	New Kingdom	Side niche burial containing pottery	OM, SEM-EDS, ATR-FTIR, GC-MS, XRD
12	Debeira East/Site 185 (Fadrus)	305:1	New Kingdom	Side niche burial containing a grindstone and pottery	OM, SEM-EDS, ATR-FTIR, GC-MS
13	Debeira East/Site 185 (Fadrus)	428:1	New Kingdom	End niche burial containing a pendant and pottery	OM, SEM-EDS, GC-MS
14	Debeira East/Site 185 (Fadrus)	512:20 (Burial B)	New Kingdom	Mud-brick chamber in pit containing two burials. Burial A had an inscribed stone fragment, a Taweret pendant, silver links, another make-up or ointment jar, a bronze bowl and pottery. Burial B was associated with pottery	OM, SEM-EDS, ATR-FTIR, GC-MS
15	Ashkeit (Site 183)	54:1	C-Group	Circular pit (adult male + adult female) containing shell beads, faience beads, carnelian beads, a scarab and a make-up applicator	OM, SEM-EDS, ATR-FTIR, GC-MS
16	Ashkeit (Site 95)	25:3a	C-Group	Oval, shallow pit containing a flexed burial with a scarab, several ostrich eggshell beads, tweezers and pottery, including one Kerma beaker	OM, SEM-EDS, ATR-FTIR, GC-MS
17	Debeira East (Site 47)	72:1	Pan-Grave	Oval pit with tumulus superstructure. Empty, except for broken Middle Kingdom style make-up container	OM, SEM-EDS, ATR-FTIR, GC-MS
18	Debeira East (Site 33)	1:5	New Kingdom	Pit burial containing an organic shroud (animal skin?) and pottery	OM, SEM-EDS, ATR-FTIR, GC-MS
19	Buhen	K10-41	Middle Kingdom	Found in Middle Kingdom layers at the New Kingdom temple	OM, SEM-EDS, ATR-FTIR
20	Buhen	K10-25	New Kingdom	Found at the New Kingdom temple	OM, SEM-EDS, ATR-FTIR
21	Debeira East/Site 185 (Fadrus)	442:2	New Kingdom	End niche burial containing faience beads, one faience pendant and one bone penannular earring	OM, SEM-EDS
22	Debeira East/Site 185 (Fadrus)	30:1	New Kingdom	Pit burial containing a feet recess and pottery	OM, SEM-EDS, XRD
23	Debeira East/Site 185 (Fadrus)	274:1	New Kingdom	Side niche burial containing a mask with traces of gold foil and blue pigment, and pottery	OM, SEM-EDS
24	Debeira East/Site 170	14:2	Pan-grave	Oval pit grave with tumulus superstructure. Empty, except for one pottery vessel and one make-up container	OM, SEM-EDS, XRD

## Materials and methods

### Sampling

Powder was scraped with a metal blade from the interior of 24 make-up containers made of various stones, except for sample 18, which was taken from an ivory container (S1 File).

### Gas chromatography-mass spectrometry

All samples were sub-sampled for analysis for both carbohydrates and lipids. The analysis method for carbohydrates is based on a published method [23]. Each sub-sample was placed in a clean 2 ml glass vial. Samples and three reference samples (acacia, tragacanth and plum gum from the reference collection at the British Museum) were hydrolysed by the addition of 500 µl of 0.5 M methanolic hydrochloric acid, heated at 80⁰C for 20 hours, and then dried under nitrogen. Samples were derivatised by the addition of 500 µl Tri-Sil HTP (hexamethyldisilazane (HMDS), trimethylsilyl chloride (TMCS), and pyridine in the ratio 2:10:1), heated at 80⁰C for 2 hours. Samples were dried under nitrogen and dissolved in 100 µl hexane in preparation for injection into the GC-MS instrument.

The GC-MS analysis was carried out with an Agilent HP5-MS column (30 m × 0.25 mm, 0.25 μm film thickness) with splitless injection, coupled to an Agilent 5973 MSD. The mass spectrometer was operating in the electron impact (EI) mode at 70 eV and scanning over the range 50–650 amu. The oven was set at 40⁰C, ramping up 9⁰C/min to 130⁰C, then 2⁰C/min to 290⁰C, held for 10 minutes.

Total ion chromatograms were extracted for ions m/z 217 and 204. Identifications of monosaccharides and acids were made by comparison of elution times with reference samples and published data [23–25]. Interpretation requires a combination of retention time and spectral features, because the epimers have near identical spectra.

The analysis method used for lipids is the standard protocol at the British Museum for unknown samples that may contain a variety of lipid molecules, such as plant oils and beeswax. It is a broad scan in order to reveal as many of these molecules as possible. Each sub-sample was placed in a clean 2 ml glass vial. Samples were solvent extracted using 1 ml dichloromethane (DCM), three times; solvent extracts were combined and dried down under a stream of nitrogen. The dried extract was derivatised using 300 µl BSTFA + 1% TMS (Sigma Aldrich 1997 BSTFA + TMCS, 99:1), heated to 70°C for 1 hour. The derivatised extracts were autoinjected into the GC-MS instrument. The GC-MS analysis was carried out with an Agilent HP5-MS column (30 m × 0.25 mm, 0.25 μm film thickness) with splitless injection, coupled to an Agilent 5973 MSD. The mass spectrometer was operating in the electron impact (EI) mode at 70 eV and scanning over the range 50–750 amu. The oven was set at 60⁰C, ramping up 10⁰C/min to 200⁰C, then 3⁰C/min to 325⁰C, held for 5 minutes. Data were interpreted using the NIST database version 2.3.

### Scanning electron microscopy-energy dispersive spectroscopy

For most samples, a Hitachi TM3000 instrument coupled with a backscattered electron (BSE) detector and an Oxford Instruments EDS was used for micromorphological examinations (SEM) and analysis of elemental composition (EDS). Powder samples were placed on a carbon tape over an aluminium stud, with no further preparation. The set-up conditions were as follows: working distance 8–12 mm, probe current 200 pA, accelerating potential 15 kV. As the samples often include a mixture of sulphides and oxides, all EDS data was quantified in both elemental and oxide forms, and normalised to 100%. Elemental data in weight percent is reported in the main paper, while stoichiometric oxides are reported in S1 File to facilitate comparisons. Spot analyses were carried out in specific grains or areas of interest identified during morphological analysis. Full-area analyses were not performed due to the complex morphology and inherent porosity of the powder samples. The identification of specific mineral phases was completed through mineralogical analysis using FTIR (see below). For each sample, images were captured at consistent magnifications (×30, × 50, × 100, × 150) to enable comparative analysis. Higher magnifications were then used to examine areas of interest in greater detail.

Samples 21, 22, 23 and 24 were analysed with a TM-1000-m-DeX instrument under low vacuum conditions,15 kV accelerating voltage, working distances 7.59 to 7.88 mm, with emission current between 36.9 and 40.1 mA. Images were acquired at magnifications of x3000, x6000, and x10000. These results were integrated in our data set, but only include major elements.

The mean chemical results reported in the paper sometimes result from the average of several grains of disparate compositions, hence they are particularly susceptible to sampling bias. However, they are deemed useful for overall comparisons. It is also worth noting that carbon and oxygen were not quantified. Detailed analyses of individual grains and areas are reported in S1 File.

### Fourier transform infrared spectroscopy

A ThermoFisher Scientific Nicolet iS5 FTIR instrument was used for all analyses, with results compared against the Kimmel standard reference library in OMNIC, as well as to previously published data [26,27]. Two different methods were employed based on the availability of samples:

1] Attenuated Total Reflectance (ATR) Mode: This method was used for all samples as it requires no sample preparation and allows for sample reuse for further analyses. ATR was employed for the general differentiation and clustering of samples. Ninety-six scans were collected at a speed of 8 cm⁻¹/s within the 4000–400 cm^-1^ range with a resolution of 4 cm^-1^. The spectra were initially acquired in transmission mode and later converted to absorbance to facilitate comparison with reference spectra from the Kimmel library and published literature.
2] Transmission Mode (KBr Pellet): For more detailed analyses of selected samples, a few milligrams were finely ground in an agate mortar, mixed with KBr powder, and pressed at 2.7 tons for 15–20 seconds to form a stable pellet. Each acquisition consisted of 64 scans recorded in the 4000–400 cm^-1^ range at 4 cm^-1^ resolution, at a speed of 8 cm⁻¹/s. resulting in well-defined spectra.

### X-ray diffraction

A Bruker D8 SMART Apex-II instrument was used to analyse samples 11, 22 and 24. Measurements were carried out using a molybdenum (Mo) anode source with characteristic wavelengths of 0.7093 Å (Kα₁) and 0.71359 Å (Kα₂). A flat graphite monochromator was employed to reduce background noise and enhance peak resolution. Data were collected using a step size of 0.06° 2θ and a step time of 720.2 seconds. The scans started at angles ranging from approximately 4.17° to 6.42° 2θ, depending on the sample. All samples were mounted in a capillary tube with sealed openings. The tube was then mounted and slowly turned (2 seconds/degree). Both the detector and radiation source were stationary. The samples were not cooled using liquid nitrogen.

## Results

All 24 samples collected were analysed using SEM-EDS. Twenty samples were analysed with ATR-FTIR, thirteen of which using FTIR in KBr transmission mode. GC-MS analysis was conducted on 18 samples, while XRD results are available for 3 samples (see Table 1 for reference).

### GC-MS

**Fig 3:**
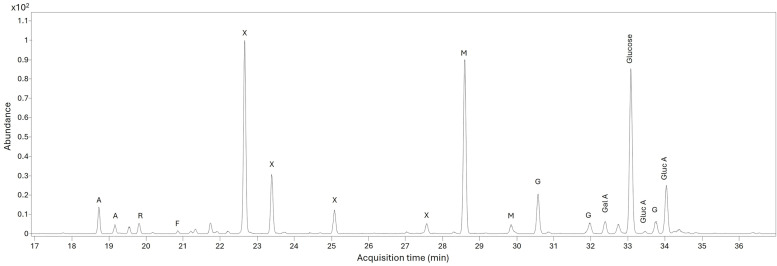
Extracted ion chromatogram (*m/z* 204 and 217) for sample 12. A = arabinose; R = rhamnose; F = fucose; X = xylose; M = mannose; G = galactose; Gal A = galacturonic acid; Gluc A = glucuronic acid.

Monosaccharides (sugars) were identified in 15 of the 18 samples (S2 File). All samples with monosaccharides present contained arabinose, and all but one contained rhamnose. Fucose was present in five samples. Xylose was present in 14 samples, often in large quantities. Mannose was present in 13 samples. Galactose was present in all samples that contained monosaccharides, as was its uronic acid. Glucose was a very major constituent of all samples that contained monosaccharides. The chromatogram obtained for sample 12 is shown in Fig 3.

**Fig 4:**
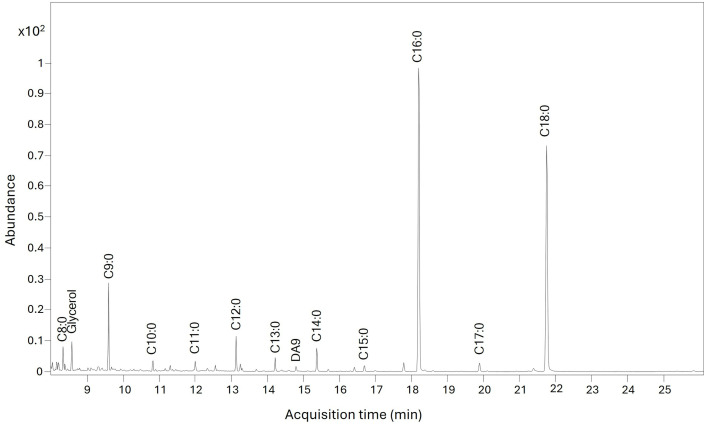
Partial ion chromatogram (8 to 26 minutes) for sample 2 showing peaks for fatty acids with carbon chain length 8 to 18. Saturation indicated by number after colon.

Lipids were identified in six samples (S2 File). Samples 3, 4 and 15 had saturated fatty acids with carbon chain length of 12, 14, 16 and 18, which shows the presence of a fatty or oily material but gives no clues as to its origin. Sample 2 had saturated fatty acids of carbon chain length 8–18 inclusive, with palmitic acid being the tallest peak (Fig 4); the presence of the odd-numbered saturated fatty acids possibly indicates an animal origin for the fatty material [28]. Sample 16 also had saturated fatty acids of carbon chain length 8–18 inclusive, and a monounsaturated fatty acid (C18:1), suggesting an animal origin, and in addition had diacids, suggesting a plant oil; altogether this suggests a mixture of fatty materials [28]. One whitish sample (18) had peaks for the monounsaturated fatty acids C16:1 and C18:1, as well as saturated fatty acids of carbon chain length 9–18, and diacids chain length 8–11. Again, this suggests a mixture of fats from both an animal and a plant origin.

### SEM-EDS

**Fig 5:**
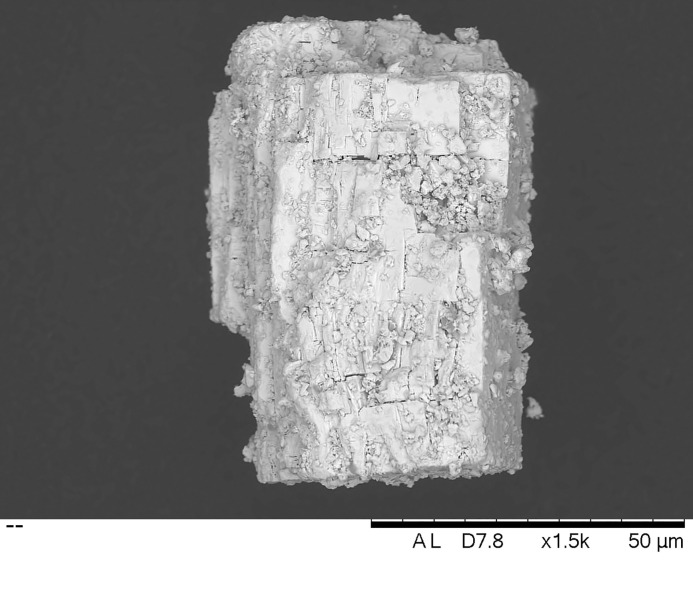
SEM-BSE image, obtained at a magnification of x1500, of a typical crystal of galena (PbS) in sample 3.

**Fig 6:**
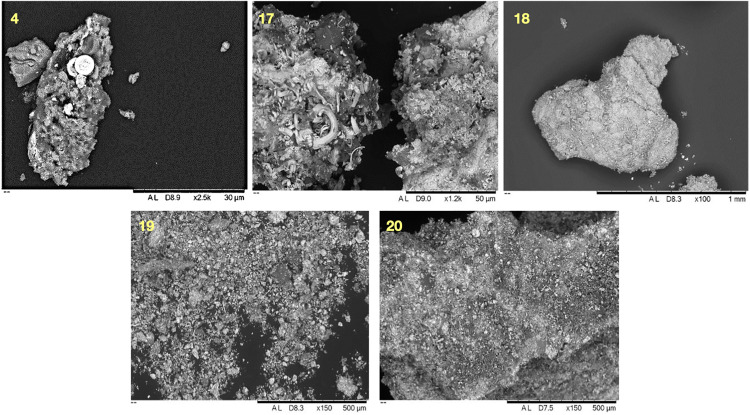
SEM-BSE images acquired at different magnifications illustrating the morphological variability of analysed samples. Circular inclusions were detected within the matrix in sample 4. Sample 17 shows plant remains intertwined in the matrix. Sample 18 shows a bright grain dominated by heavy metals. Samples 19 and 20 show mixtures of grains of different sizes, morphologies and compositions.

The microanalytical study of samples shows that the majority of the samples are dark grey or black, and include distinctive grains of galena (Fig 5), i.e., a bluish-grey cubic mineral with a metallic lustre consisting of lead sulphide [29]. However, this occurs in different forms and combinations. For example, samples 3, 9, 10, 13, 14 and 15 display mixtures of discrete mineral grains, whereas other samples resemble conglomerates of more heterogeneous composition with various minerals embedded within. Fibrous structures, perhaps of plant origin, were detected in samples 4, 8, 10 and 14. Samples 4 and 17 also showed peculiar lenticular grains of possible biogenic origin. Both traits could be characteristic of the Debeira region, although further analyses are needed. Sample 12 presented a yellowish shade, different from the others, whereas samples 18, 19 and 20 were whitish, different both in terms of colour and morphology from all other samples (Fig 6).

In agreement with previous reports, SEM-EDS analysis showed Pb and S as the dominant elements in virtually all samples (with a few exceptions discussed below) (Table 2). Illustrative examples of this are samples 3, 5, 15 and 16.

**Table 2:** The elemental composition of the 24 kohl samples obtained with the EDS detector. Further information reported in S1 File.

SAMPLE	Na	Mg	Al	Si	P	S	Cl	K	Ca	Ti	Mn	Fe	Cu	Zn	Sb	Ba	Pb
1	1.8	0.7	3.6	8.6	0.8	2.3	0.3	0.7	9.1	0.1	0.1	4.8	4.3	4.8	0.1	0.5	**57.4**
2	1.8	0.3	1.1	1.9	0.2	9.0	0.9	0.5	3.3	–	0.4	1.2	2.8	5.2	–	0.1	**71.5**
3	1.2	0.1	1.1	0.5	0.1	6.7	2.1	0.4	0.2	–	0.1	0.2	2.4	3.1	–	0.2	**82.1**
4	1.1	0.2	0.6	1.7	0.2	15	3.3	0.1	5.2	0.1	0.1	0.9	1.4	5.4	0.2	–	**64.8**
5	0.3	0.1	0.5	0.9	0.3	9.0	0.2	0.1	1.0	–	0.5	1.2	1.7	2	0.1	0.1	**82.2**
6	1.3	0.2	1.2	2.1	0.1	4.4	1.7	0.2	2.2	–	15.0	1.5	5.0	2.9	0.1	1.2	**59.7**
7	2.5	0.4	0.3	0.4	19.7	3.1	1.1	–	60.5	–	0.7	0.4	5.4	4.5	0.6	0.2	**19.7**
8	2.2	1.0	4.2	12.3	1.3	4.6	0.7	0.8	16.3	0.1	–	4.6	7.5	4.4	0.4	0.5	**39**
9	3.2	0.3	3.7	11.1	0.2	7.4	4.0	1.1	4.8	–	0.1	2.8	4.9	3.9	0.5	0.9	**52.1**
10	4.9	0.3	1.6	5.0	0.1	0.1	–	0.3	2.8	–	–	7.2	3.6	8.6	0.1	0.3	**59.4**
11	1.6	0.2	1.3	5.8	0.7	9.0	0.8	0.3	2.3	–	–	1.1	3.1	4.9	0.1	0.4	**68.3**
12	1.9	1.5	5.0	15.9	0.6	1.9	0.1	0.7	18.7	0.6	0.5	5.5	5.4	5.2	31.2	0.2	**7.4**
13	3.5	3.4	8.1	52.3	0.5	2.5	0.2	1.0	5.5	–	0.2	7.8	6.4	6.0	0.5	0.7	**0.5**
14	1.8	1.6	4.6	12.5	0.5	2.4	4.6	0.7	7.3	0.4	0.1	5.4	3.6	3.7	0.4	0.4	**50.7**
15	2.4	0.3	0.3	0.4	–	10.3	–	0.1	0.5	–	0.1	0.6	2.9	15.1	–	–	**67.0**
16	1.7	0.2	0.8	9.0	0.1	7.8	1.4	0.4	1.9	–	–	6.6	4.9	4.5	0.1	0.1	**60.8**
17	1.2	1.9	9.3	31.0	2.2	3.5	1.8	1.6	12.9	1.4	–	9.2	3.3	2.1	0.3	0.3	**19.9**
18	0.6	2.8	13.4	30.0	0.6	7.6	0.3	0.8	26.8	4.9	–	1.3	0.2	3.5	0.6	2.6	**4.2**
19	0.6	0.5	1.7	6.4	0.9	7.0	7.7	0.5	8.0	0.3	–	3.4	–	–	–	–	**63.3**
20	0.7	0.6	1.1	4.1	0.1	10.9	6.5	3.4	5.0	0.4	–	4.0	–	3.8	–	–	**59.5**
21	–	–	5.6	23.9	–	2.9	–	–	5.6	–	–	–	–	–	–	–	**62.2**
22	–	–	–	–	–	9.4	–	–	–	–	–	–	–	–	–	–	**90.6**
23	–	–	–	–	–	11.3	–	–	–	–	–	–	–	–	–	–	**88.7**
24	–	–	–	1.4	–	17.6	0.5	–	2.2	–	–	–	–	9.9	–	–	**65.9**

However, notable variation within the corpus of Pb-rich samples was detected. A heavy element detected in the majority of the analysed samples is Zn, often (but not always) accompanied by Cu. Purer samples 10, 15 and 24 included a higher amount of Zn, but this element is also present in more diluted samples (see below). This stands in contrast with the only occasional occurrence of Zn compounds in Egyptian make-up substances, especially kohls [19]. While zinc sulphides or sulphates can occur naturally in association with lead minerals, we cannot rule out the possibility that these might have been deliberately added, in some cases at least, to lighten the colour of the cosmetic substance. Zinc and lead sulphides occur together, alongside magnetite and pyrite, in gold bearing strata in the Egyptian and Sudanese Eastern Desert [30,31]. The higher frequency of Zn in Nubian make-up in comparison with Egyptian samples is suggestive of distinct sources.

In addition to the heavy elements, and to variable extents, virtually all samples also contained lighter minerals and compounds, typically rich in Ca, Si, and Al, but also showing notable concentrations of Na, Mg and other elements. In order to estimate the composition of these inorganic additives or accessory ingredients, the average chemical compositions were re-normalised after neglecting all elements heavier than Fe, in addition to S (most frequently present in sulphides) and Cl (most likely reflecting the deposition of salts during weathering) (Table 3). The resulting table also includes a column for the ‘original sum’, providing a crude indication of the total weight of lighter compounds in each sample. The results reinforce the recurrent presence of Si, Al and Ca, suggesting that clay and/or calcareous materials were included, perhaps to lighten the colour of the make-up substance and/or adjust other properties. Sample 9, for example, includes clear fragments of quartz and aluminosilicates mixed with pure galena grains, and samples 14 and 17 show discrete calcite fragments. In other cases, the mixtures appear more finely intertwined, although it is not clear whether this reflects a geological association or finer grinding and mixing with an organic binder. Mg and Na may have been impurities in the geological ingredients employed, although the latter may also result from the post-depositional crystallisation of salts. It should be noted that floods have frequently compromised the state of preservation of material culture in Lower Nubia [21].

**Table 3:** Re-normalised composition of samples 1–21 excluding heavy elements, S and Cl. Samples 22–24 did not include light elements in significant concentrations.

SAMPLE	Na	Mg	Al	Si	P	K	Ca	Ti	Mn	Fe	Original sum
**1**	6	2	12	28	3	2	30	0	0	16	30
**2**	17	3	10	18	2	5	31	0	4	11	11
**3**	31	3	28	13	3	10	5	0	3	5	4
**4**	11	2	6	17	2	1	51	1	1	9	10
**5**	6	2	10	18	6	2	20	0	10	24	5
**6**	5	1	5	9	0	1	9	0	63	6	24
**7**	3	0	0	0	23	0	71	0	1	0	85
**8**	5	2	10	29	3	2	38	0	0	11	43
**9**	12	1	14	41	1	4	18	0	0	10	27
**10**	22	1	7	23	0	1	13	0	0	32	22
**11**	12	2	10	44	5	2	17	0	0	8	13
**12**	4	3	10	31	1	1	37	1	1	11	51
**13**	4	4	10	64	1	1	7	0	0	9	82
**14**	5	5	13	36	1	2	21	1	0	15	35
**15**	51	6	6	9	0	2	11	0	2	13	5
**16**	8	1	4	43	0	2	9	0	0	32	21
**17**	2	3	13	44	3	2	18	2	0	13	71
**18**	1	3	17	37	1	1	33	6	0	2	81
**19**	3	2	8	29	4	2	36	1	0	15	22
**20**	4	3	6	21	1	18	26	2	0	21	19
**21**	0	0	16	68	0	0	16	0	0	0	35

Sample 7 stands out in that it is nominally pure calcium phosphate, with some surface contamination of Pb. The sample was taken from the lip of a travertine (CaCO_3_) container that was heavily eroded due to floodings at the site, which potentially contributed to the adsorption of phosphate on the stone surface [32,33]. Sample 8, extracted from the inside of the same vessel, yielded results that are more consistent with the majority of Pb-rich samples.

**Fig 7:**
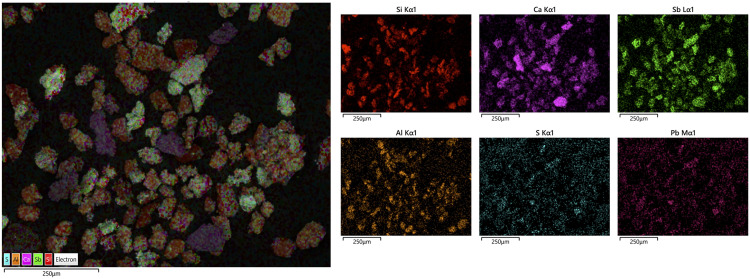
SEM-EDS mapping of sample 12. Si, Al and Ca are the dominant elements in the matrix, indicating the presence of clay in the mixture. However, Ca-bearing Sb-rich particles are also clearly discernible, consistent with the presence of calcium antimonate in the sample.

**Fig 8:**
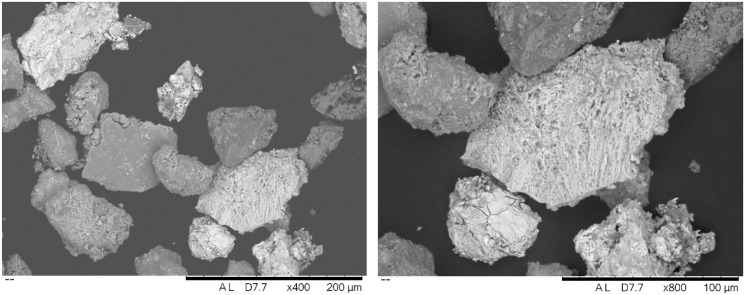
SEM-BSE images of sample 12 at magnifications of x400 and x800, illustrating the characteristic morphology of crystals of calcium antimonate (bright particles) among other components.

Of particular interest is sample 12, dominated by Ca and Sb – a combination never reported for archaeological cosmetic substances. Microscopic examination and X-ray elemental distribution maps show general enrichment of Si, Al, Ca and Pb, consistent with the average compositions (Fig 7). However, besides the addition of clay to the mixture (indicated by concentrations of Si and Al), our analysis also detected particles of pure calcium antimonate (Ca_2_Sb_2_O_7_). Additionally, the small concentrations of Pb and S likely suggest galena contamination. Higher magnification observation shows the Sb-rich crystals to have a fibrous texture, made up of long acicular crystals (Fig 8).

Lead and calcium antimonate are artificially synthesised compounds used, respectively, as yellow and white colourants and opacifiers in ancient Egyptian glass-making from the New Kingdom or Late Bronze Age [34]. Lead antimonate was occasionally detected in Egyptian make-up substances before, having been usually interpreted as an impurity of galena ores [18,35]. Previous studies suggested that calcium antimonate crystallised *in situ* after the addition of a Sb-bearing compound to a batch of glass [34]. However, more recent studies emphasising the artificial production of calcium antimonate indicate that it was deliberately added to glass [36]. The first identification of calcium antimonate as the main ingredient in Nubian cosmetics, as reported here, further demonstrates that this compound was purposefully synthesised and also used outside the glass industry in the ancient Nile valley.

**Fig 9:**
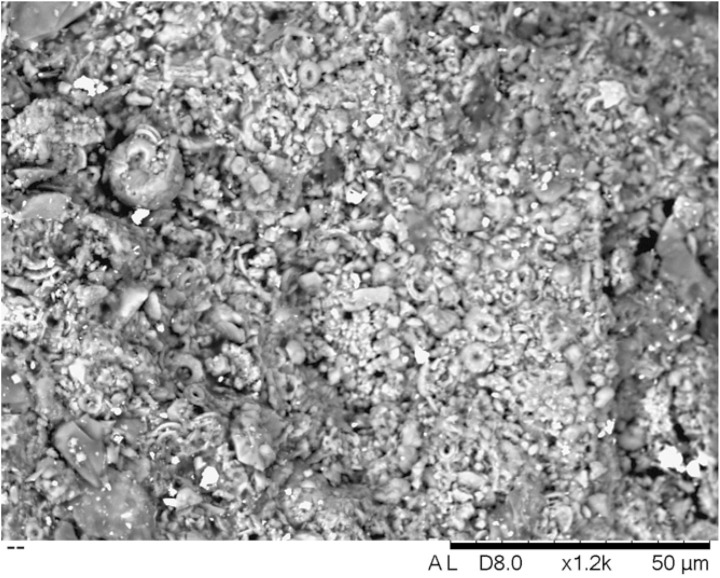
SEM-BSE image of sample 18 at ×1200 magnification, showing microfossils in the clay-like matrix.

Another distinctive case is sample 18. Similar to the previous sample, PbS is largely absent here, with only a few scattered grains detected, possibly resulting from contamination during grinding. However, a more heterogeneous, aluminium-rich matrix is observed, along with scattered signals of Ba (as baryte), Zn, feldspar inclusions, and other grains rich in Mg and Si clusters. These features suggest the presence of kaolinite—a whitish clay abundant in Sudan and found in Kushite mortars [37,38]. Combined with the predominant presence of calcareous microfossils, this could explain the distinct shade observed in this sample (Fig 9). Baryte and zinc-bearing minerals can occur as accessory components of lead ores, but they also occur in association with gold-bearing quartz veins in the Eastern Desert of both Egypt and Sudan [39,40]. The distinctive identification of these compounds suggests that baryte was procured independently and used separately. Further analysis to identify the microfossils present in the sample may reveal paleoenvironmental information, shedding further light on the potential source of the raw materials [41–43].

**Fig 10:**
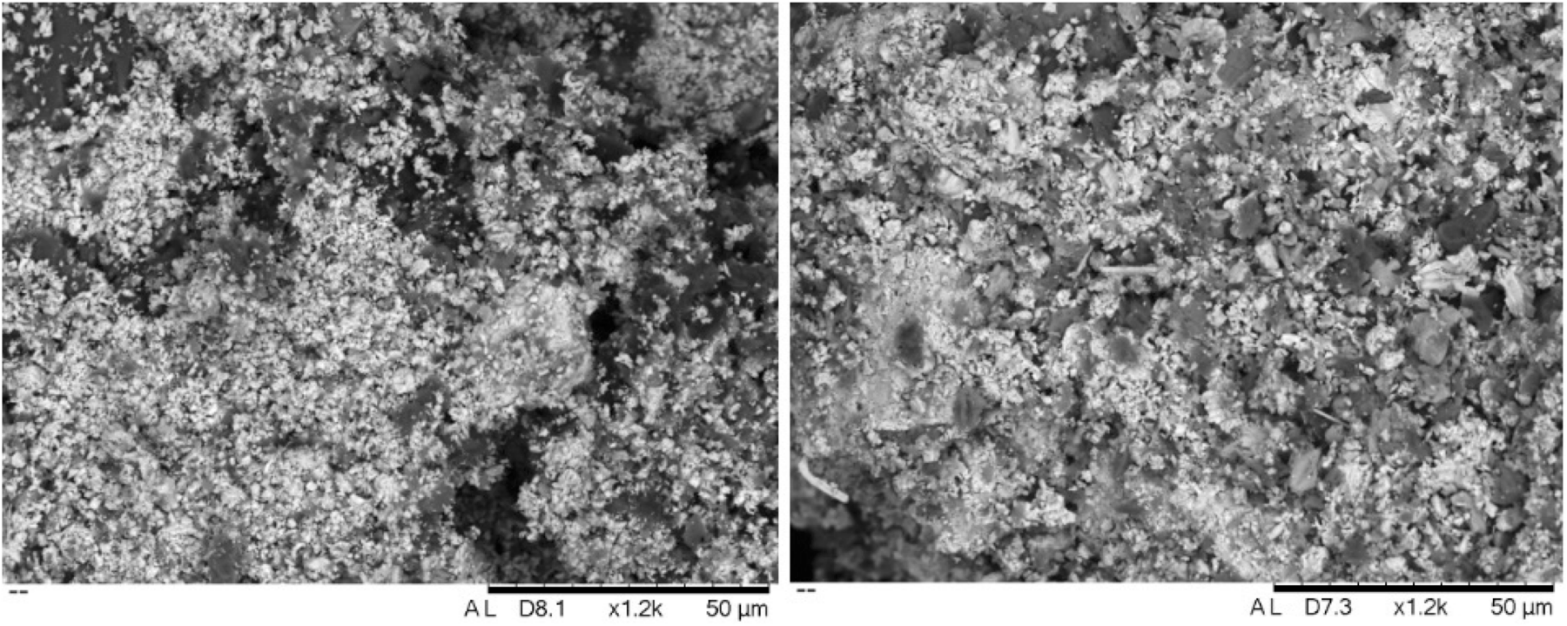
SEM-BSE images of samples 19 (left) and 20 (right) at ×1200 magnification. Both samples display a very similar morphological composition, yet form a distinct cluster compared to the remaining samples. This suggests a specific make-up recipe, likely linked to the use of different raw materials—such as gypsum—identified through their morphology and confirmed by SEM-EDS analysis.

**Fig 11:**
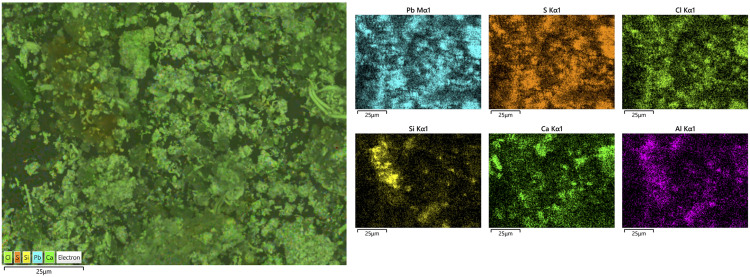
SEM-EDS mapping of sample 19. Al, Si, and Ca clusters indicate a clay-based bulk composition. Pb appears as the dominant element, consistent with the presence of galena. However, unlike earlier samples, S is also detected at high levels in Ca-rich particles, pointing to the presence of gypsum in the mixture.

Samples 19 and 20, from Buhen, are also lighter-coloured mixtures that stand out in their microstructure and composition (Fig 10). While the composition of the samples is dominated by Pb, and some PbS inclusions are identified in X-ray maps, galena crystals are not so clear or conspicuous (Fig 11). It is possible that the material is dominated by a lighter-coloured lead compound, such as cerussite (PbCO_3_) or anglesite (PbSO_3_), both previously reported for Egyptian make-up recipes [19]. Association between Pb and Cl is also suggestive of other weathering products of Pb-rich minerals; e.g., cotunnite, which has been reported for Egypt [19]. In addition, the recurrent association between Ca and S is consistent with the presence of gypsum, which was confirmed by FTIR.

### ATR-FTIR

**Fig 12:**
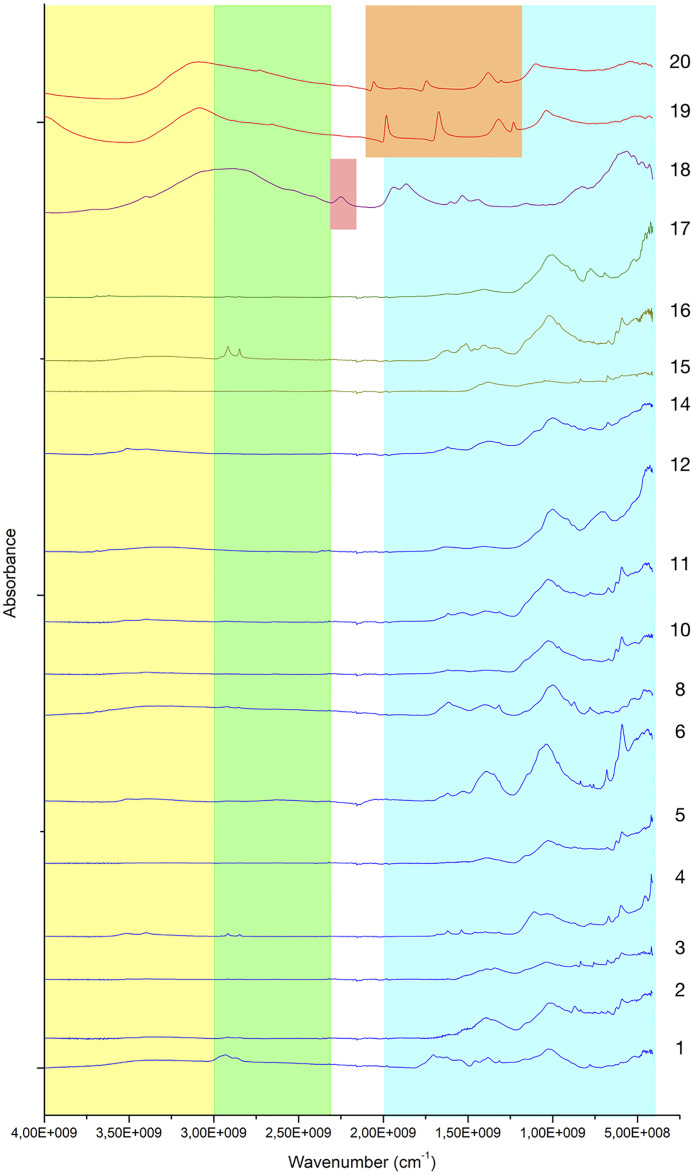
Stack of ATR-FTIR spectra for all analysed samples. Colours are used to identify the three ranges employed to differentiate molecular compounds [26]. Yellow: 4000-3000 cm^-1^ range; green: 3000 to 2400 cm^-1^ range; blue: 2000 to 500 cm^-1^ range. Samples from Fadrus, Askheit and Debeira do not display major differences, while the compositions of samples 18 (Debeira/Site 33), 19 and 20 (Buhen) appear as significant outliers.

ATR-FTIR spectroscopy was used to confirm and expand the phase identifications suggested by SEM-EDS examination, in addition to helping with the grouping of samples based on similarities in their infrared spectra (S3 Table). A comparison of spectra reveals significant variability (Fig 12; Table 4).

**Table 4:** Peaks identified in the ATR-FTIR spectra. Samples are color-coded by site: blue for Fadrus (site 185), green for Ashkeit (Site 183), yellow for Debeira Site 47, purple for Debeira Site 33, and red for Buhen. While some clustering and similarities are observed across sites (such as samples 1-2-3, samples 10−11 and samples 19−20), notable variability across the dataset is also attested. All values are reported in cm^-1^.

Sample	2930−2840	1630−1520	1390−1370	1320−1310	1050−1020	780−750	690−660	Extra Peaks
1	x	x	x	x	x	x		1704, 1666, 1457
2	x		x		x	x	x	
3	x		x		x	x	x	975, 866, 835, 626, 591
4	x	x					x	3525, 3407, 1108, 874, 596, 435
5			x				x	969, 874, 840, 623, 591
6		x	x		x	x	x	836, 590
8	x	x		x		x		1403, 1000, 873, 577
10		x		x	x	x	x	963, 624, 591
11		x		x	x	x	x	969, 876, 592
12								1001, 915, 870, 711
14		x	x	x		x	x	3512, 3395, 1000, 924, 870,
15			x		x		x	870, 836
16	x	x			x		x	1508, 1400, 966, 837, 591
17	x					x	x	1004, 876
18	x	x			x	x	x	3531, 3401, 1736, 1419, 873, 799
19	x	x				x	x	3518, 3401, 1682, 1507, 1338, 1111, 835, 647, 593
20	x	x				x	x	3528, 3397, 1682, 1507, 1338, 1090, 835, 595

To better identify the molecular composition of these samples, additional FTIR analyses using the KBr pellet method were carried out. In these analyses (), a clay matrix was recognised by the characteristic Si–O stretching band of clay at 1035 cm^-1^, with all samples displaying this peak sharply within a narrow range of 1030–1039 cm^-1^. Additionally, a peak at 1080 cm^-1^ corresponding to the inherent SiO₂ quartz structure was observed. Two other silica bands in the 798–780 cm^-1^ range further confirm the presence of quartz, which allows us to describe this matrix as a quartz-rich clay.

**Fig 13:**
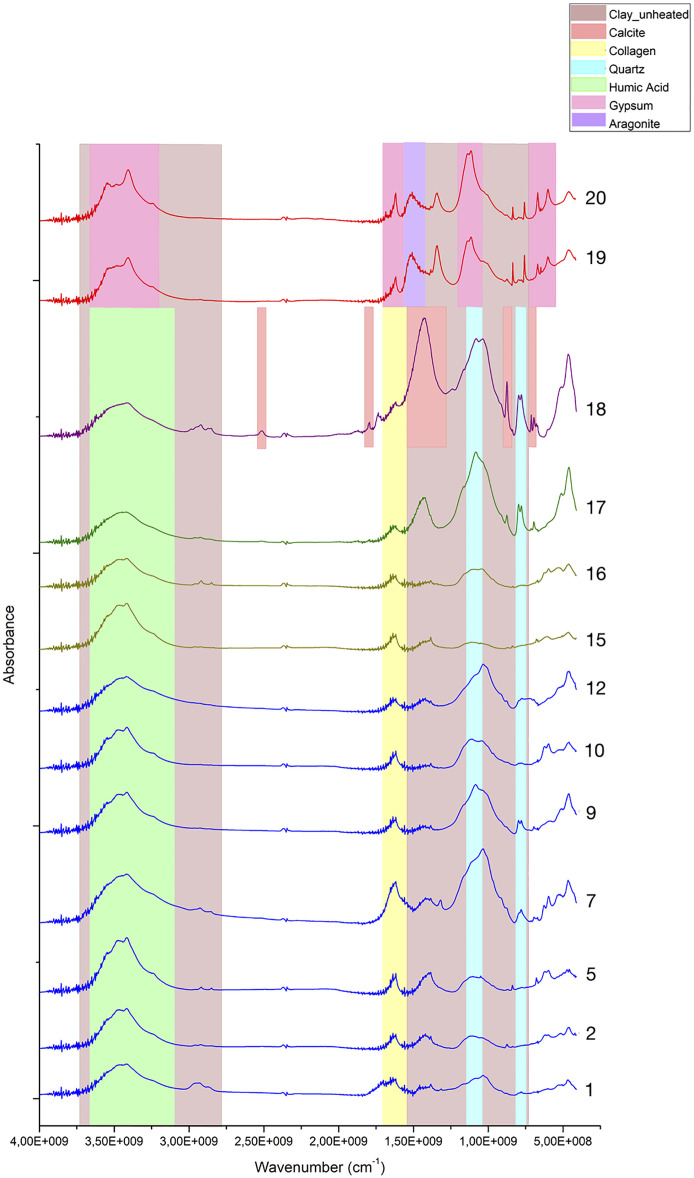
Stacked FTIR spectra illustrating the molecular variability of samples analysed using the KBr pellet technique. A consistent feature across all samples is the presence of a quartz-rich clay (indicated in brown). Collagen was also detected in the majority of the analysed samples. Sample 18 shows the presence of calcite. Samples 19 and 20 exhibit a significantly higher proportion of gypsum and aragonite compared to clay, indicating a potential change in the production process of these make-up mixtures.

**Fig 14:**
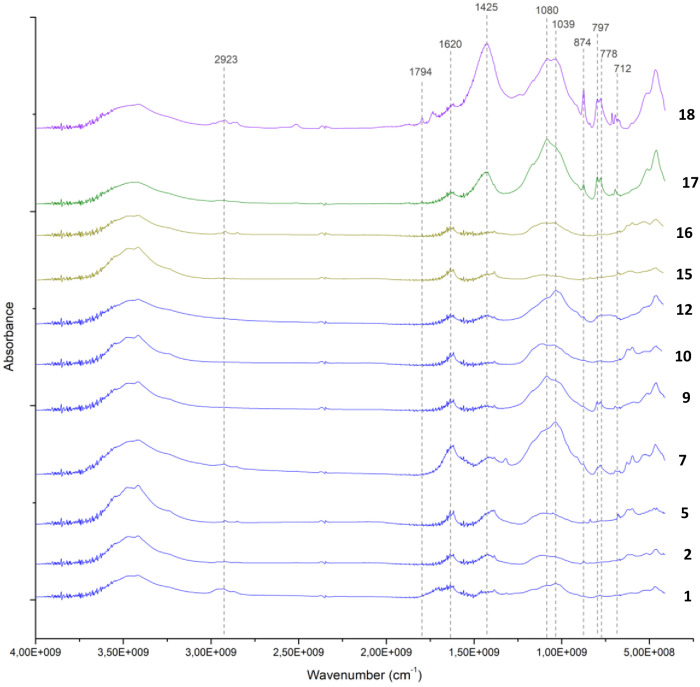
FTIR spectra of analysed samples showing the range between 4000 cm^-1^ to 500 cm^-1^. The characteristic peak of unheated clay at 1039 cm^-1^ and quartz at 1080 cm^-1^ and 797−778 cm^-1^ are recognisable in all the samples. A difference appears in sample 18, in which calcite is detected at 1794 cm^-1^, 1425 cm^-1^, 874 cm^-1^ and 712 cm^-1^.

**Fig 15:**
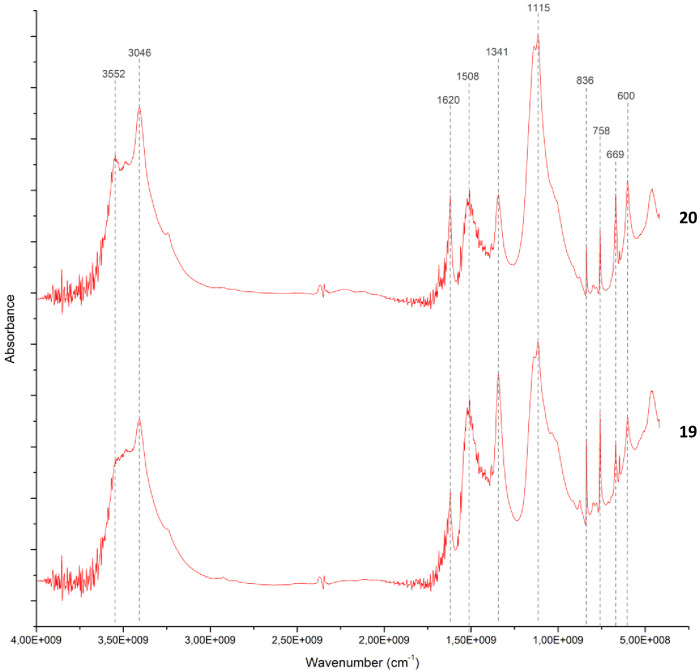
FTIR spectra of analysed samples from Buhen showing the range between 4000 cm^-1^ to 500 cm^-1^. The characteristic peaks of gypsum at 3404 cm^-1^, 1620 cm^-1^, 1145 cm^-1^, 1115 cm^-1^ and 669 cm^-1^ are visible. Moreover, aragonite was also detected in these samples, with a characteristic peak at 1508 cm^-1^.

An unheated clay matrix was identified in all analysed samples. Indeed, the position and definition of the SiO stretching band are closely linked to firing conditions [44–46]. In unheated clay, this band is detected around 1035 cm^-1^, shifting to 1042 cm^-1^ at approximately 700°C, and splitting between 1050–1078 cm^-1^ at 800°C. The band further shifts to 1082 cm^-1^ at 900°C. These changes correspond to the gradual breakdown of the clay structure, formation of amorphous phases, and recrystallisation of new minerals. In the analysed samples, the SiO peak consistently appears around 1039 cm^-1^, indicating the absence of significant exposure to heat or low-temperature treatment [47–50].

Moreover, at c. 1620 cm^-1^, peaks of collagen were detected for most samples, confirming the addition of animal fat to various mixtures, as also detected by GC-MS. The identification of animal fat in these cosmetics paves the way for palaeoproteomic analyses to provide further detail. At c. 3500 cm^-1^, the presence of humic acid suggests organic contamination.

**Fig 16:**
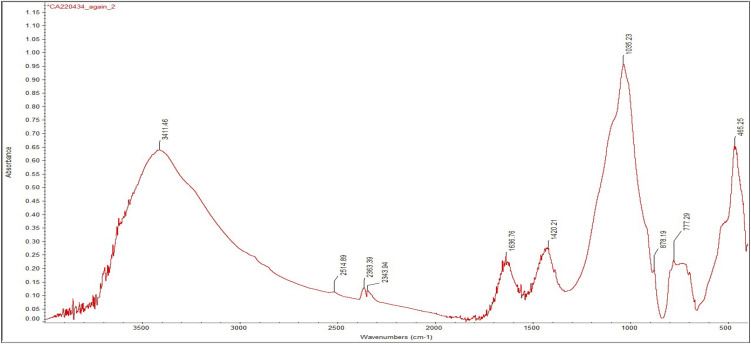
Spectrum obtained for sample 12 identifying calcium antimonate.

Despite similarities, FTIR analysis also revealed greater variability, allowing us to identify different recipes used in the production of make-up substances. Sample 12 is again significant in this regard. The presence of calcium antimonate could be observed. Calcium antimonate is more suitable for detection with FTIR than galena, due to the presence of oxygen and the possibility of IR-active modes. The latter is an oxide mineral, and oxides often have characteristic absorption bands in the infrared region—in this case, SbO and CaO. The obtained spectrum shows the characteristic clay peak at 1035 cm^-1^, but the characteristic peaks of calcite at 1420 cm^-1^ and 878 cm^-1^ are also present (Fig 16). Moreover, the characteristic peaks of SbO (antimonates) are recognised between 900−600 cm^-1^ [51].

**Fig 17:**
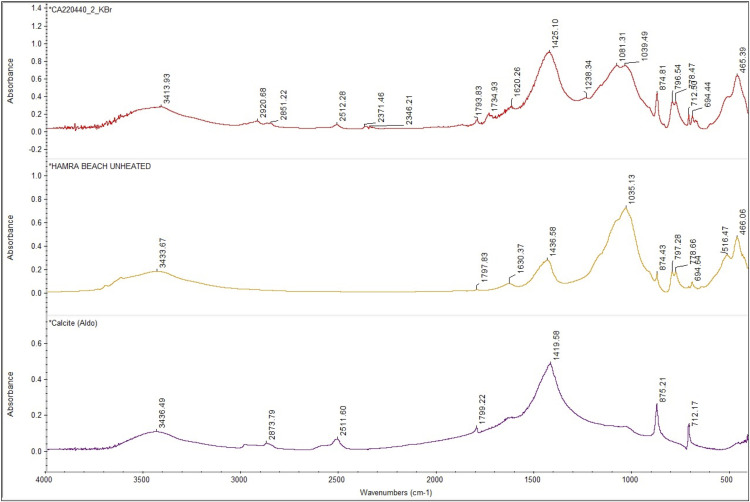
Comparison of the FTIR spectrum obtained for sample 18 and reference data in the Kimmel online library.

**Fig 18:**
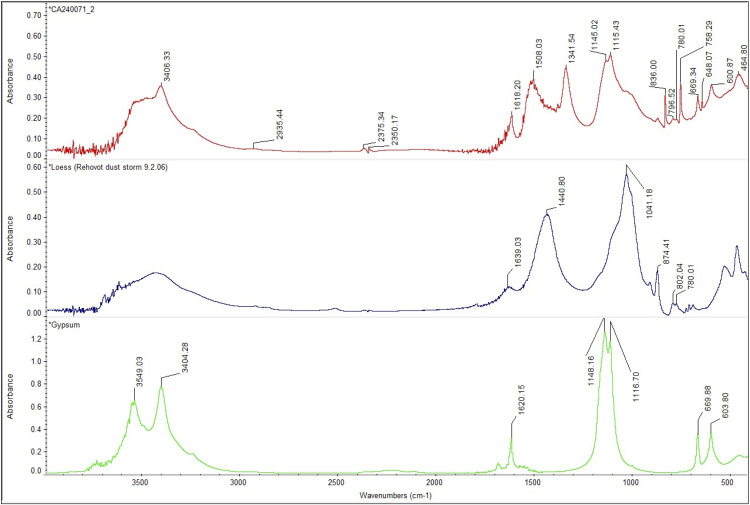
Comparison of the FTIR spectrum obtained for sample 19 and reference data in the Kimmel online library.

The presence of calcite at 1420, 875 and 712 cm^-1^ in sample 18 indicates a clay matrix rich in calcareous components (Fig 17). Analysis of samples 19 and 20 also revealed the use of a clay matrix, but mixed with gypsum (Fig 18), identified by peaks 3404 cm^-1^, 1620 cm^-1^, 1145 cm^-1^, 1115 cm^-1^and 669 cm^-1^. Despite the absence of calcite, aragonite (CaCO_3_) was also detected (at 1508 cm^-1^), which might be an impurity or suggest the presence of shells in the matrix.

### XRD

Samples 11, 22 and 24 were analysed with XRD. Samples 11 and 22 consisted of a black powder, while sample 24 consisted of a lighter-coloured powder, similar to the samples 19 and 20. The main phases of samples 11 and 24 were identified as Pb_2_O(SO_4_) and PbS, which is consistent with the lead-dominated nature of the black powder samples.

**Fig 19:**
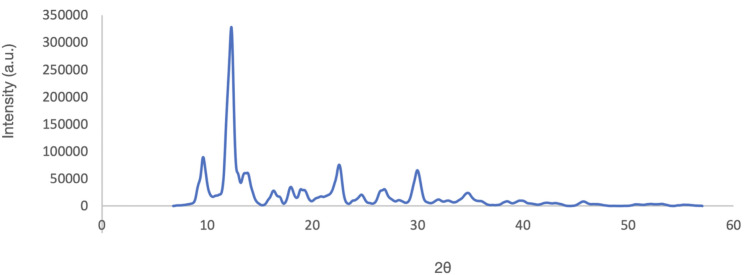
Diffractogram for sample 22, in which SiO_2_ was identified as major phase.

Analysis of sample 22 revealed a different composition. According to its diffractogram, SiO_2_ constitutes its major phase (Fig 19). This would be comparable to sample 15, which also contains S and Zn, though in different concentrations. Recent analyses of Egyptian make-up recipes also revealed silica-based mixtures [19].

## Discussion

The analysis of make-up samples from Sudanese Lower Nubia reported here has shed light on different mixtures used to produce a variety of products. Both plant and animal fats were mixed with galena and other mineral components to produce make-up in Bronze Age Nubia, which is supported by Egyptian textual sources [52].

It has been suggested that the plant gums most likely to have been available to inhabitants of the ancient Nile valley were acacia (gum Arabic), fruit gums, and tragacanth [53]. Tragacanth would have been imported, possibly from Syria, Turkey or Iran [53], whereas acacia and fruit gums were available locally in Nubia [54].

Interpretation of gum spectra from ancient samples is not straightforward because it is unknown how the original gum may have deteriorated over the last c. 3000 years and in the various environments to which samples were historically subjected. However, the presence of certain monosaccharides can suggest plant origins for the gum. All but one sample contained mannose, which is found in fruit gum but not in tragacanth or acacia, and five samples contained fucose which is found in tragacanth but not in acacia or fruit gum [24]. This indicates that a mixture of gums may have been used.

Previous analyses of monosaccharides from ancient Egyptian material have produced similar gum profiles, with what appears to be a mixed plant origin [55]. The high prevalence of glucose is frequently found in archaeological samples, and is likely a contaminant from packing materials and the general atmosphere; the high levels of xylose may also be explained this way [24].

Evidence for lipids was found in six of the samples. In three, the fatty acids were not distinctive enough to be able to identify the origin of the fatty materials, but suggest the use of plant oil or animal fat as a binder for the mineral powder. The presence of diacids in two samples may indicate a plant origin. However, both these samples also had odd carbon number fatty acids and monounsaturated fatty acids, which suggest an animal fat origin. It is always possible for archaeological samples that any lipids could be from contamination, from the burial environment, from handling, from transportation. However, these samples were taken from areas unlikely to have been frequently handled, and only 6 contained lipids, despite similar burial environments and storage conditions, which suggests that the lipids were added to the original material as a binder. This may indicate that a mixture of fatty materials was used. One sample had saturated fatty acids of both even and odd numbers, which may indicate an animal origin.

Analytical results of the inorganic components show that galena (PbS) was the most widespread material in the production of make-up products used in Sudanese Lower Nubia, which mirrors the situation in Egypt [14,19]. The common high concentration of Zn and Cu are likely ore impurities, although the high Zn concentrations in some samples might be suggestive of the deliberate addition of Zn compounds. Recent Pb isotopic analysis of some of the samples analysed here revealed two clearly distinguishable sources. One of these could be identified as the galena mines of Gebel el-Zeit in Egypt’s Eastern Desert [56], while the other remains unidentified. Importantly, Zn and Cu are present in samples from both isotopic groups, therefore these elements alone do not provide useful information regarding provenance.

In samples predominantly characterised by PbS as major component, the occasional high concentration of Al and Si point to the use of clay as matrix for ground galena, which would probably not have altered its visual effect. However, significant outliers were identified, suggesting local sociocultural dynamics and the intention to create distinctive visual effects on the body, particularly those achieved with lighter-coloured products. The small concentration of PbS in some of these outliers is likely due to grinding practices resulting in the mixture of various substances [56].

It is important to note that light-coloured make-up substances have also been detected for Egypt, including Pb-bearing minerals like cerussite, but also alternative components such as calcites. However, over 90% of analysed Egyptian samples consist of kohl—black galena-based make-up—with only c. 7% of analysed samples consisting of green (atacamite or malachite, not attested in Sudanese Nubia) and whitish/light brownish make-up substances not containing Pb [19]. While the significance of these outliers amid a large corpus of analysed samples is difficult to assess for Egypt, the situation seems to be different for Sudanese Nubia, where the contextualization of archaeological and scientific data might point to local cultural practices.

**Fig 20:**
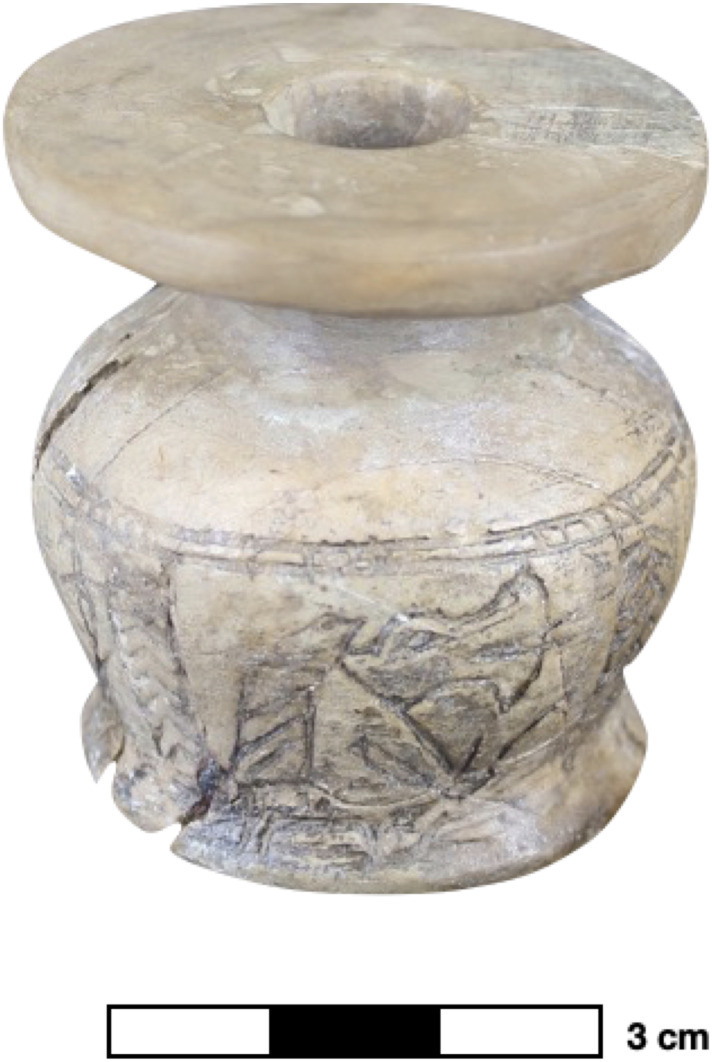
An ivory kohl container from a pit burial in Debeira East/Site 33 [21]. Photo by R. Lemos, courtesy of Gustavianum, Uppsala University Museum. The decoration of the container is consistent with Kerma decorative patterns, especially the representation of goddess Tawaret [58].

Galena was not employed in the manufacture of the make-up product extracted from the only ivory container in the data set (sample 18). The main component of the sample was clay with a high percentage of calcareous microfossils. Besides the light-coloured make-up substance inside, the decorated ivory container expresses local cultural affiliations. Ivory was probably the most culturally charged material used in Nubia since the Neolithic to manufacture various objects, especially jewellery [9]. More specifically, the decoration of the container establishes links with Kerma (Fig 20) [57], which are also suggested by the archaeological context of the grave where it was excavated (Table 1). The association of a rare ivory container with an exceptionally distinctive, light-coloured make-up substance likely unveils local Nubian bodily ideals, which might have been connected to local cultural expressions.

Samples 19 and 20 also presented a different composition. Although galena was detected in these samples, the addition of gypsum would have produced a different visual effect on the body, which is suggested by the light-coloured powder extracted from these containers. Together with sample 18, these samples potentially indicate a distinctive Nubian tradition of body decoration.

The identification of calcium antimonate in Nubia make-up products further suggests the intention to achieve a distinctive visual impact with light-coloured make-up products. Calcium antimonate is an artificially synthesised substance achieved by heating antimonate oxides and calcium carbonates at temperatures around 1100^o^C [36]. The resulting calcium antimonate was used as a colourant and opacifier in the Egyptian glass industry from the 18th Dynasty [34], while its use outside glass-making in the Nile valley has remained unknown until now. The use of calcium antimonate in make-up products from Sudanese Lower Nubia highlights the deliberate intent to produce light-coloured make-up substances. This differs from the situation in Egypt, where the composition of occasionally used light-coloured substances consists of minerals that occur naturally with galena. The use of calcium antimonate in make-up substances in Sudanese Nubia also suggests the high level of technological skill available locally. This is especially significant since calcium antimonate has never been detected in the large corpus of analysed samples from Egypt.

## Conclusion and future research

The first extensive analysis of make-up samples from Bronze Age contexts in Nubia allows insight into the internal diversity of cosmetic mixtures in Nubia, as well as notable differences with the assortment of recipes recorded in Egypt [19].

We demonstrated the presence of plant gum and animal fat as binding materials in make-up products from Bronze Age Nubia. The profile of the gum, although difficult to interpret, indicates a mixed plant origin, and is similar to gum profiles from ancient Egypt. The fatty acid profiles in two cases seem to indicate a mixture of animal and plant fat/oil. Such mixtures have also been detected in samples from Egypt [19]. However, no terpenoids or aromatics were found in the cosmetic products in this study, which might point to cultural differences across the ancient borders of Egypt and Nubia. Paleoproteomic analysis will likely shed more light on the animal fats used in these make-up mixtures.

The results of this study support earlier assumptions about the predominance of galena in make-up products, which can therefore be classified as kohl [14]. However, at the same time, this study supports more recent suggestions of a greater diversity of make-up mixtures in ancient Egypt, while considerably expanding our knowledge by including samples from Sudanese Lower Nubia. Despite similarities with the Egyptian corpus, the significant outliers identified in the corpus from Sudan likely point to cultural variability across the borders of ancient Egypt and Nubia.

To this day, this remains the most extensive exploration of kohl samples across the entire Nile valley. By including, for the first time, Lower Nubian samples from Sudan, our study opens new research avenues into make-up production and use in ancient Northeast Africa. The first identification of calcium antimonate and clay-based mixtures, designed to achieve different visual effects on the body, significantly enhances our understanding of the diverse cosmetic practices in ancient Northeast Africa—far beyond what the study of ancient Egyptian make-up products alone can reveal.

## Supporting information

S1 File Catalogue of sampled objects and SEM data. (DOCX)

S2 File GC-MS data. (DOCX)

S3 Table ATR-FTIR peaks. (XLSX)

S4 File Abstract in Arabic. (DOCX)
