# Supplementary material for: Bronze Age make-up recipes from Sudanese Lower Nubia point to a greater diversity across cultural borders in ancient Northeast Africa
Source: PLoS One. 2025 Sep 11;20(9):e0330205. doi: 10.1371/journal.pone.0330205 (PMC12425255; doi:10.1371/journal.pone.0330205)
Supplement: S1 File — (DOCX) [file pone.0330205.s001.docx]

**Bronze Age make-up recipes from Sudanese Lower Nubia point to a greater diversity across cultural borders in ancient Northeast Africa**

Rennan Lemos,^1*^ Caterina Zaggia,^1^ Kate Fulcher,^2^ Einar Lidén,^3^ Ludmila Werkström,^4^ Emma Hocker,^4^ Jonas Bergquist,^3^ Marcos Martinón-Torres^1^

^1^ Department of Archaeology and McDonald Institute for Archaeological Research, University of Cambridge, UK

^2^ Institute of Archaeology, University College London, UK

^3^ Department of Chemistry, Uppsala University, Sweden

^4^ Gustavianum, Uppsala University Museum, Sweden

^*^ Corresponding author: [rdsl3@cam.ac.uk](mailto:rdsl3@cam.ac.uk)

**Supporting Information 1: Catalogue of sampled objects and SEM-EDS data (Ox%)**

**Sample 1 (200:7; CA220423)^[[1]](#footnote-1)^**


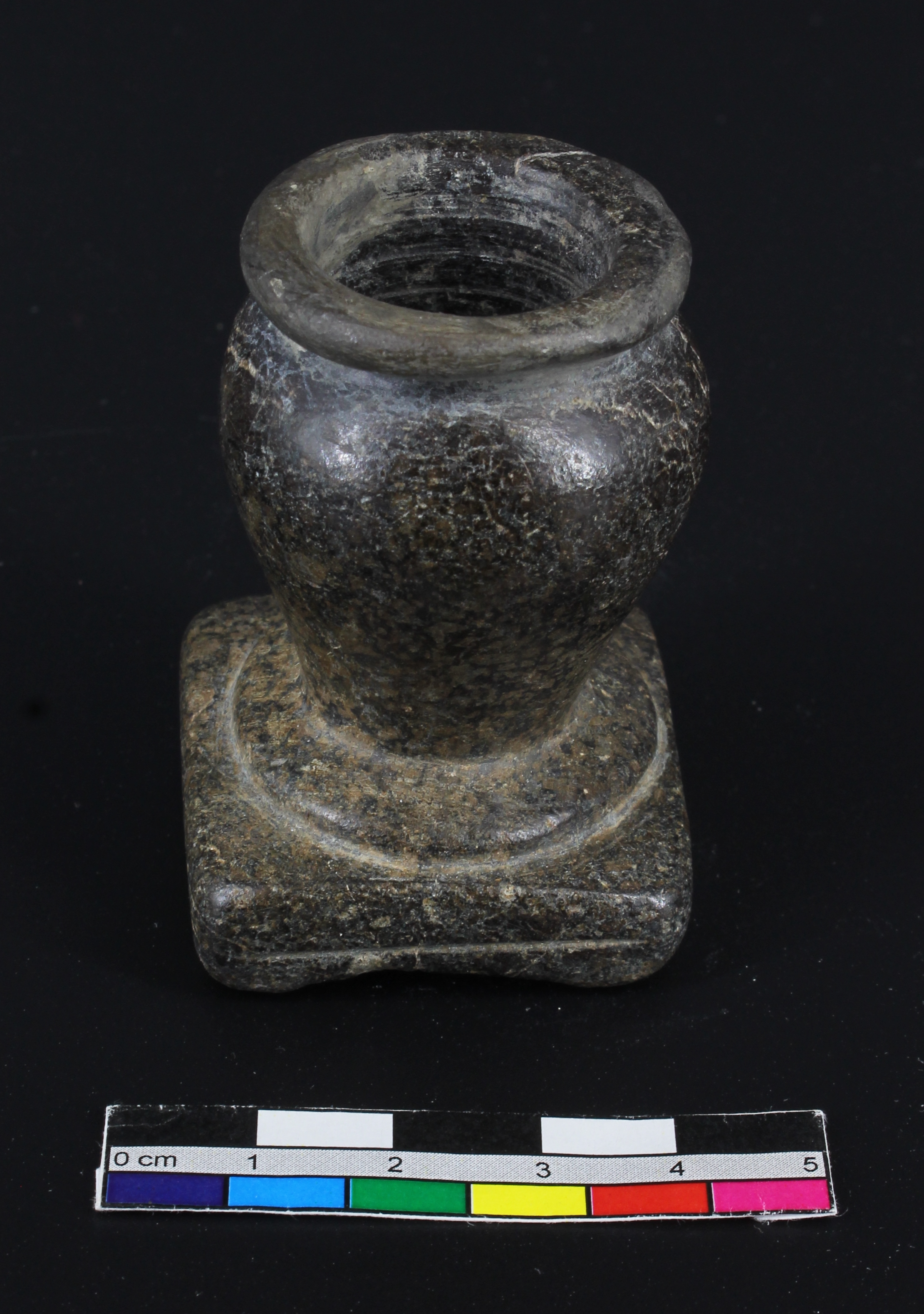


Figure 1: kohl container from Debeira East/Site 185 (200:7) (Säve-Söderbergh and Troy 1991). Photo by R. Lemos. Courtesy of Gustavianum, Uppsala University Museum.


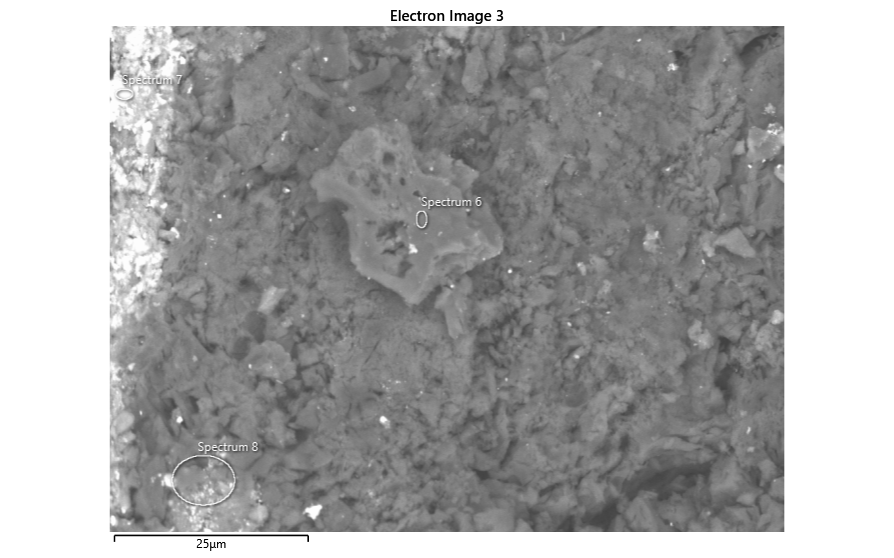


Figure 2: SEM image showing reading spots.

| Spectra | Na_2_O | MgO | Al_2_O_3_ | SiO_2_ | P_2_O_5_ | SO_3_ | Cl | CaO | FeO | CuO | PbO |
| --- | --- | --- | --- | --- | --- | --- | --- | --- | --- | --- | --- |
| Spot 6 | 2.6 | 0.4 | / | 1.6 | / | 2.0 | 0.6 | 10.5 | / | / | 82.4 |
| Spot 7 | 0.3 | 0.3 | 1.0 | 2.1 | / | 9.5 | / | 2.1 | 1.4 | / | 83.4 |
| Spot 8 | 0.9 | 0.6 | 4.2 | 9.9 | 1.6 | 1.5 | 0.8 | 7.1 | 4.8 | / | 67.4 |

Table 1: elemental composition.


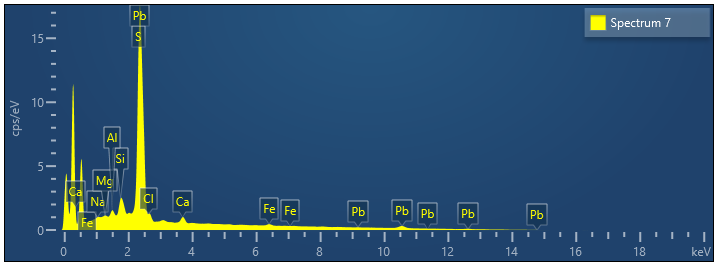


Figure 3: spectrum of spot 7 highlighting PbS as major component.

**Sample 2 (2:1; CA220424)**


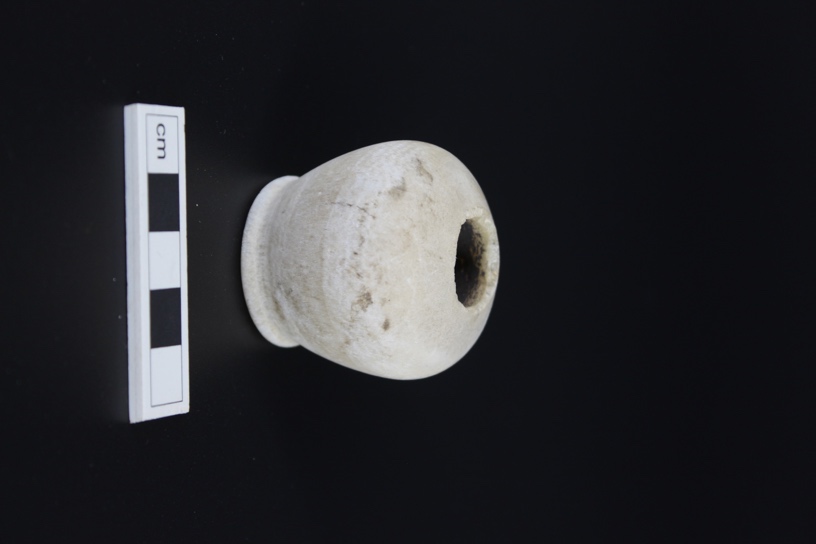


Figure 4: kohl container from Debeira East/Site 185 (2:1) (Säve-Söderbergh and Troy 1991). Photo by R. Lemos. Courtesy of Gustavianum, Uppsala University Museum.


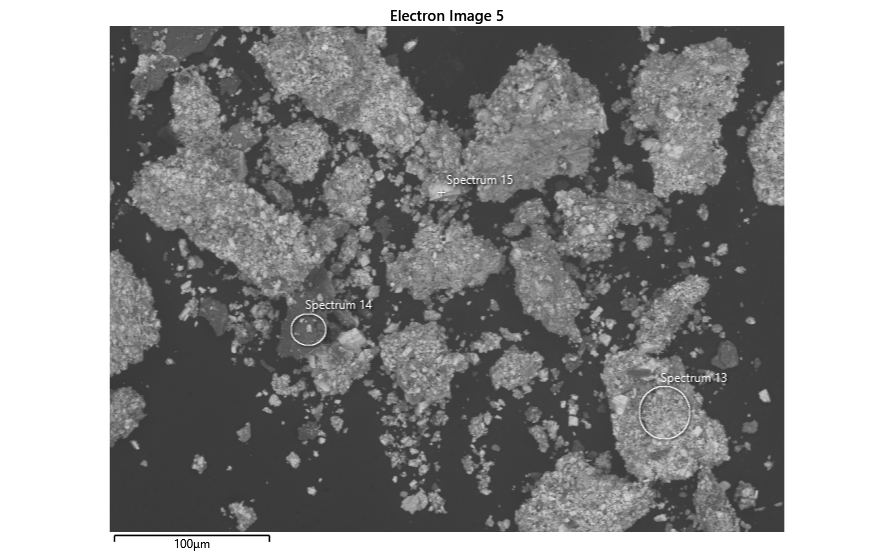


Figure 5: SEM image showing reading spots.

| Spectra | Na_2_O | MgO | Al_2_O_3_ | SiO_2_ | SO_3_ | Cl | K_2_O | CaO | FeO | ZnO | PbO |
| --- | --- | --- | --- | --- | --- | --- | --- | --- | --- | --- | --- |
| Spot 13 | 0.5 | 0.3 | 0.9 | 2.1 | 7.5 | 0.8 | / | 3.3 | 1.7 | 2.8 | 80.2 |
| Spot 14 | 0.7 | 2.3 | 1.5 | 3.3 | 2.0 | 0.4 | 0.6 | 66.1 | 1.5 | / | 21.6 |
| Spot 15 | / | / | 0.3 | / | 11.5 | / | / | 0.4 | / | / | 87.7 |

Table 2: elemental composition.


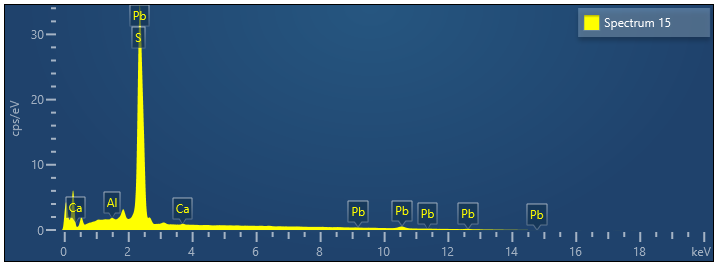


Figure 6: spectrum of spot 15 showing PbS as major component.

**Sample 3 (22:1; CA220425)**


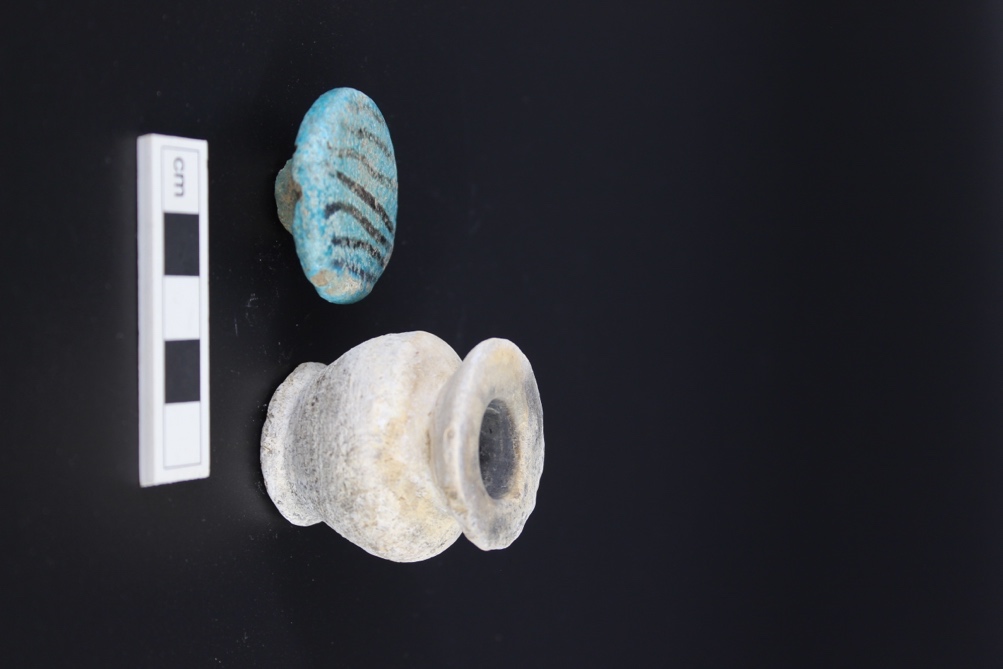


Figure 7: kohl container from Debeira East/Site 185 (22:1) (Säve-Söderbergh and Troy 1991). Photo by R. Lemos. Courtesy of Gustavianum, Uppsala University Museum.


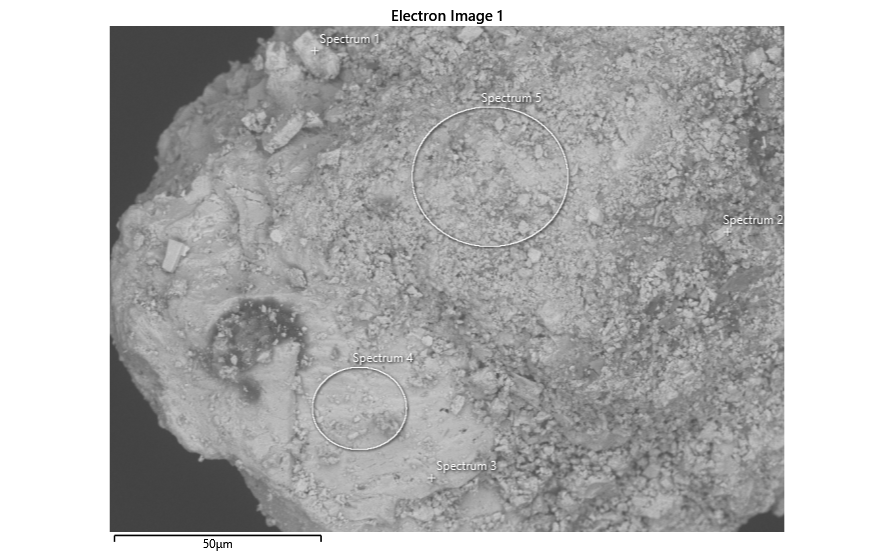


Figure 8: SEM image showing reading spots.

| Spectra | Na*_2_*O | MgO | Al_2_O_3_ | P_2_O_5_ | SO_3_ | Cl | CaO | ZnO | SnO | PbO |
| --- | --- | --- | --- | --- | --- | --- | --- | --- | --- | --- |
| Spot 1 | / | / | 0.4 | / | 10.9 | / | / | / | / | 88.7 |
| Spot 2 | / | / | 6.9 | / | 4.1 | 3.8 | / | 3.5 | / | 81.8 |
| Spot 3 | / | / | 0.5 | 0.9 | 1.4 | 4.1 | 0.4 | 3.5 | / | 89.2 |
| Spot 4 | 0.4 | 0.1 | 0.5 | / | 1.5 | 8.5 | / | / | / | 89.1 |

Table 3: elemental composition.


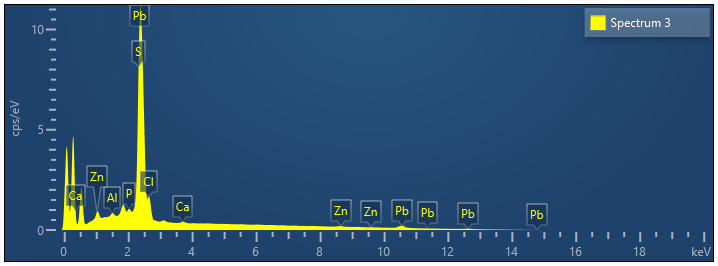


Figure 9: spectrum of spot 3 showing PbS as major component.

**Sample 4 (56:2; CA220426)**


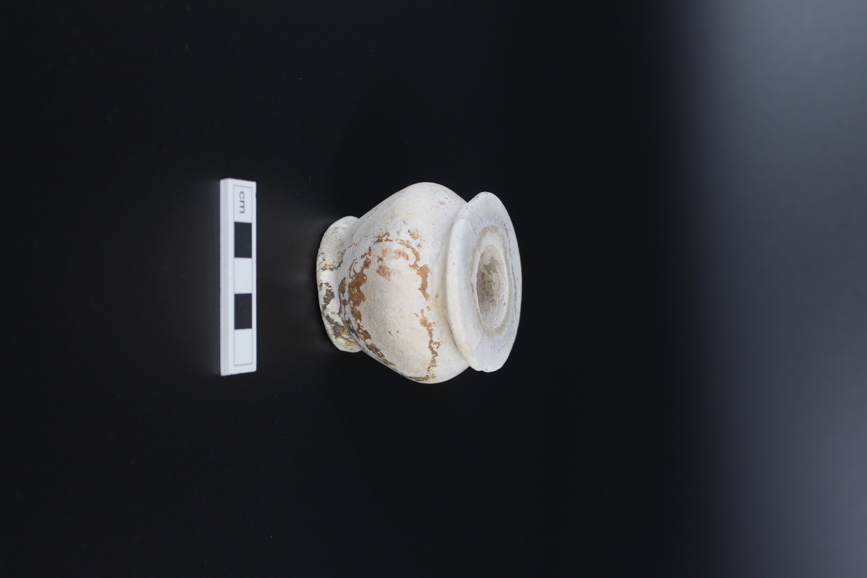


Figure 10: kohl container from Debeira East/Site 185 (56:2) (Säve-Söderbergh and Troy 1991). Photo by R. Lemos. Courtesy of Gustavianum, Uppsala University Museum.


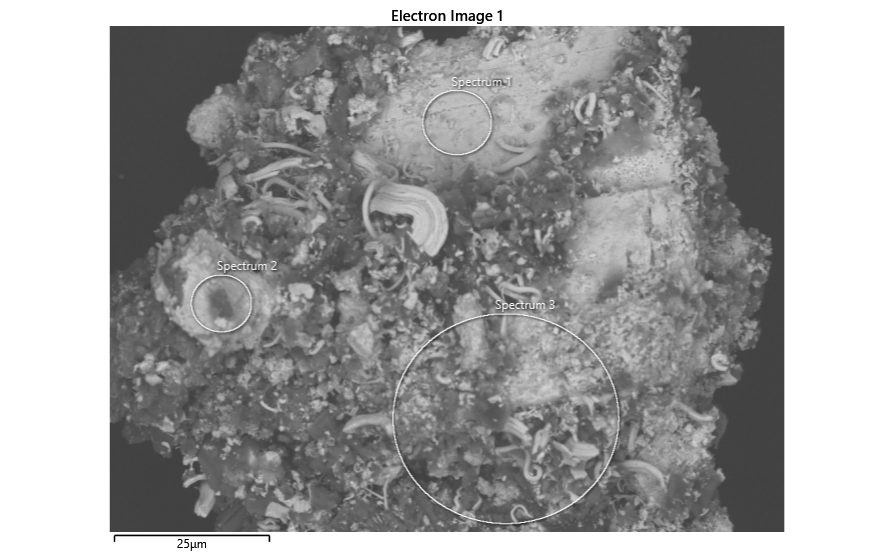


Figure 11: SEM image showing reading spots.

| Spectra | Na_2_O | MgO | Al_2_O_3_ | SiO_2_ | SO_3_ | Cl | CaO | FeO | ZnO | SnO | PbO |
| --- | --- | --- | --- | --- | --- | --- | --- | --- | --- | --- | --- |
| Spot 1 | 0.4 | / | 0.3 | / | 11.0 | 2.9 | 0.9 | / | / | / | 85.6 |
| Spot 2 | 0.4 | / | 0.5 | 0.9 | 12.4 | 4.1 | 8.9 | / | 1.7 | / | 71.2 |
| Spot 3 | 0.3 | 0.2 | 0.5 | 1.5 | 10.1 | 7.2 | 11.5 | 1.1 | 2.6 | 0.3 | 64.8 |

Table 4: elemental composition.


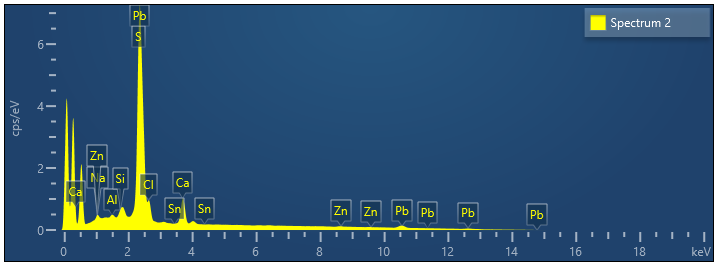


Figure 12: spectrum of spot 2.

**Sample 5 (73: 2; CA220427)**


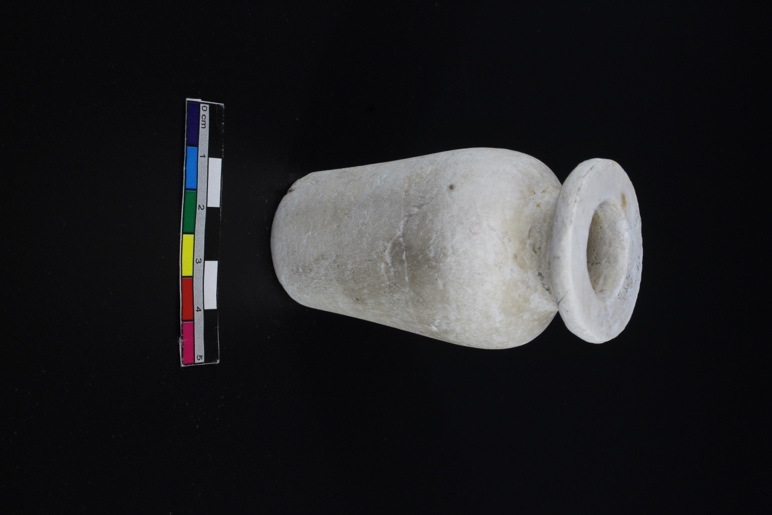


Figure 13: kohl container from Debeira East/Site 185 (73:2) (Säve-Söderbergh and Troy 1991). Photo by R. Lemos. Courtesy of Gustavianum, Uppsala University Museum.


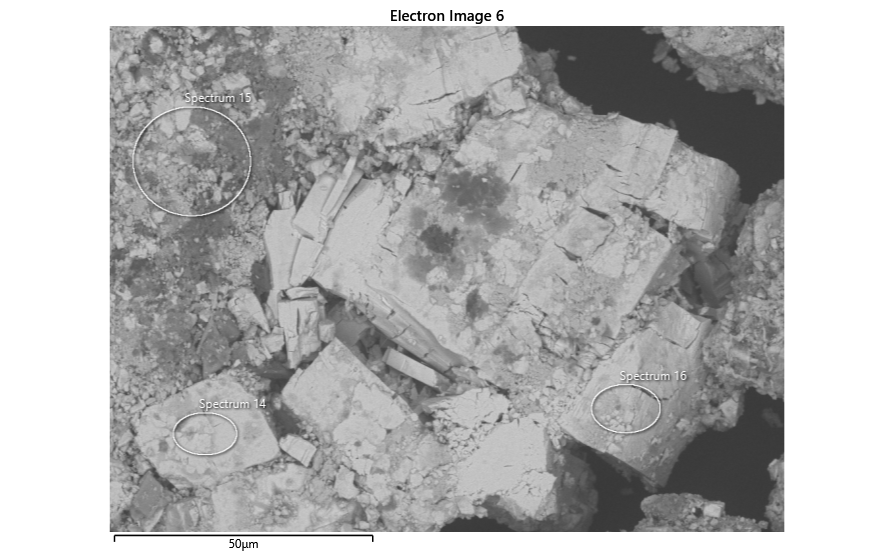


Figure 14: SEM image showing readings spots.

| Spectra | Na_2_O | MgO | Al_2_O_3_ | SiO_2_ | SO_3_ | CaO | MnO | FeO | ZnO | SnO | TiO_2_ | PbO |
| --- | --- | --- | --- | --- | --- | --- | --- | --- | --- | --- | --- | --- |
| Spot 14 | 0.4 | 0.2 | 0.3 | 0.5 | 12.4 | / | / | / | / | 0.4 | / | 85.7 |
| Spot 15 | / | 0.6 | 0.5 | 0.9 | 6.4 | 0.5 | 1.7 | 10.0 | 3.6 | / | 0.4 | 55.0 |
| Spot 16 | 0.2 | / | 0.3 | 0.4 | 8.7 | 0.3 | 0.6 | / | / | / | / | 70.1 |

Table 5: elemental composition.


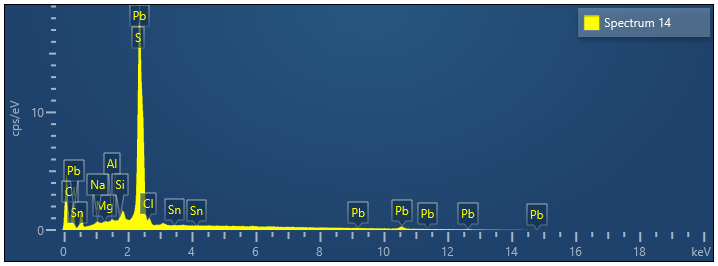


Figure 15: spectrum of spot 14.

**Sample 6 (84:33; CA220428)**


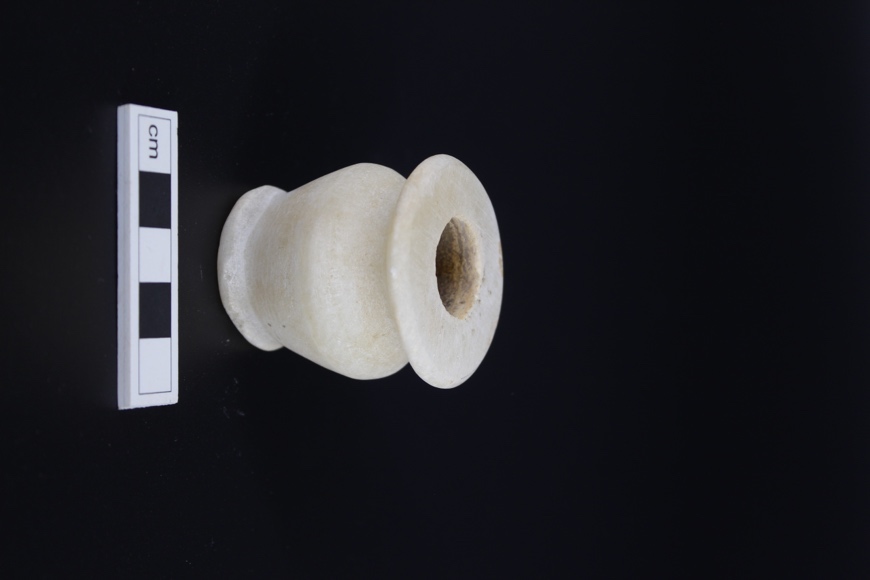


Figure 16: kohl container from Debeira East/Site 185 (83:44) (Säve-Söderbergh and Troy 1991). Photo by R. Lemos. Courtesy of Gustavianum, Uppsala University Museum.


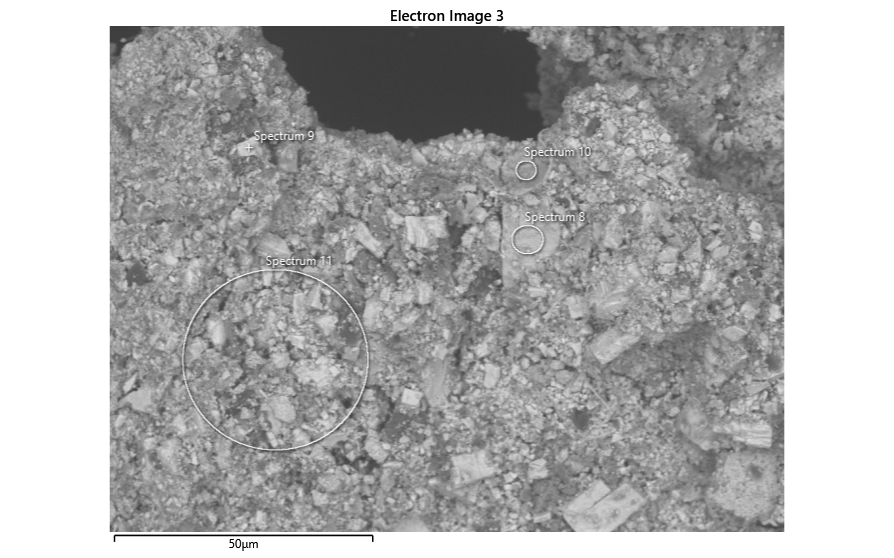


Figure 17: SEM image showing reading spots.

| Spectra | Na_2_O | Al_2_O_3_ | SiO_2_ | P_2_O_5_ | SO_3_ | Cl | K_2_O | CaO | MnO | FeO | SnO | PbO |
| --- | --- | --- | --- | --- | --- | --- | --- | --- | --- | --- | --- | --- |
| Spot 8 | 0.3 | 0.6 | 0.8 | / | 12.4 | / | / | 0.7 | 0.8 | / | / | 84.6 |
| Spot 9 | 0.4 | 1.2 | 1.0 | / | 11.1 | / | / | 1.0 | 0.9 | 0.6 | / | 83.8 |
| Spot 10 | 0.3 | 0.7 | 2.6 | 2.4 | 5.3 | 2.9 | 0.3 | 4.4 | 2.8 | 1.1 | / | 77.5 |
| Spot 11 | 0.5 | 1.7 | 4.8 | / | 9.4 | / | / | 2.9 | 4.6 | 1.8 | / | 74.3 |

Table 6: elemental composition.


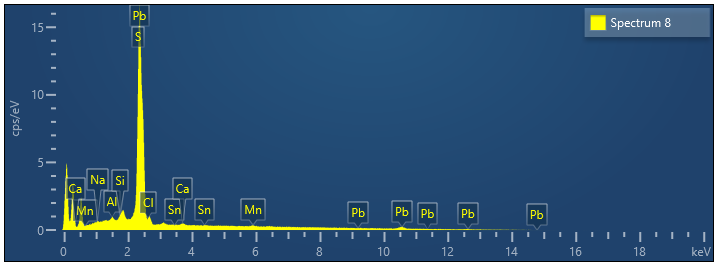


Table 7: spectrum of spot 8.

**Sample 7 (128:2; CA220429)**


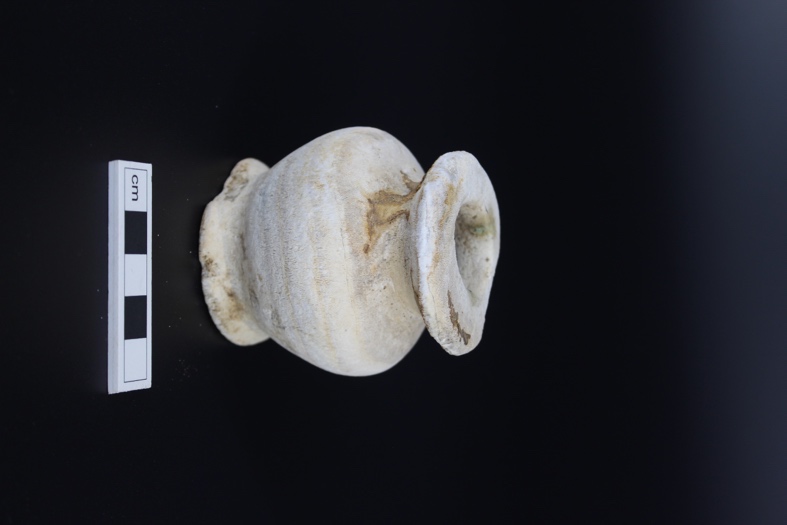


Figure 18: kohl container from Debeira East/Site 185 (128:2) (Säve-Söderbergh and Troy 1991). Photo by R. Lemos. Courtesy of Gustavianum, Uppsala University Museum.


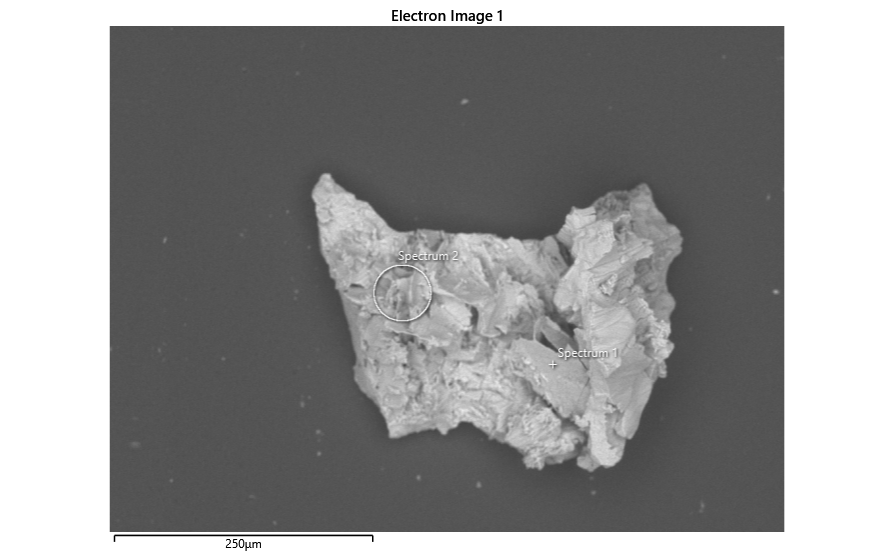


Figure 19: SEM image showing readgin spots.

| Spectra | Na_2_O | MgO | P_2_O_5_ | SO_3_ | Cl | CaO | MnO | CuO |
| --- | --- | --- | --- | --- | --- | --- | --- | --- |
| Spot 1 | 1.0 | 0.8 | 29.3 | 2.4 | 1.5 | 64.6 | 0.5 | / |
| Spot 2 | 1.1 | 0.5 | 24.1 | 2.1 | 1.3 | 69.2 | 0.5 | 1.1 |

Table 8: elemental composition.


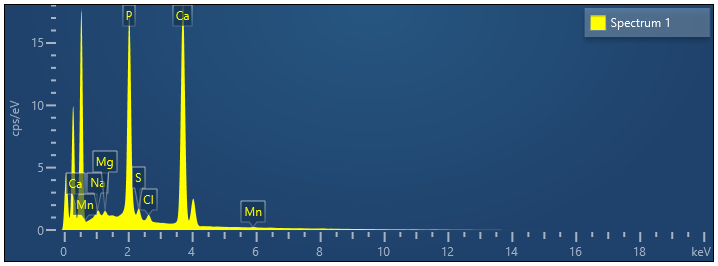


Figure 20: spectrum of spot 1.

**Sample 8 (128:2; CA220430)**


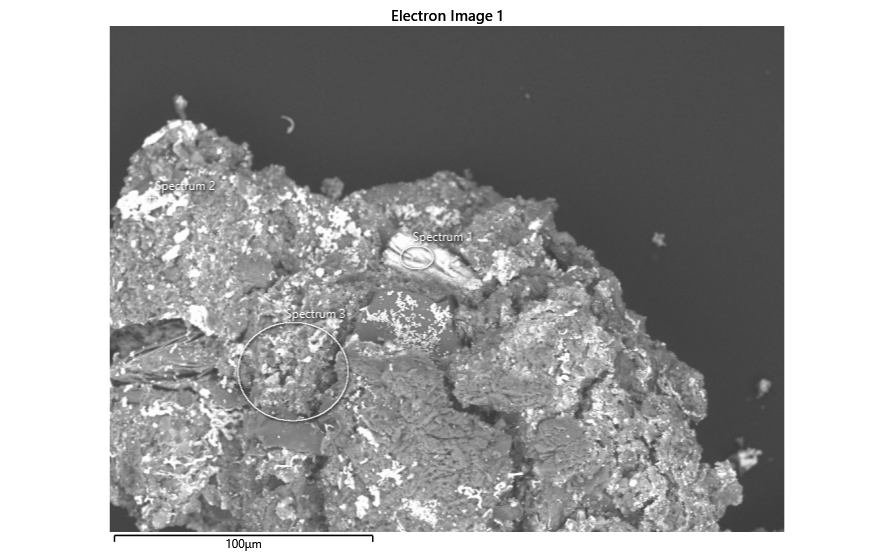


Figure 21: SEM image showing reading spots.

| Spectra | MgO | Al_2_O_3_ | SiO_2_ | P_2_O_5_ | SO_3_ | Cl | K_2_O | CaO | FeO | CuO | PbO |
| --- | --- | --- | --- | --- | --- | --- | --- | --- | --- | --- | --- |
| Spot 1 | / | 0.9 | 2.1 | 1.0 | 9.4 | / | 0.4 | 5.0 | 1.1 | 1.0 | 79.2 |
| Spot 2 | / | 1.7 | 4.5 | / | 9.6 | / | / | 3.7 | 1.6 | 0.2 | 78.8 |
| Spot 3 | 1.6 | 7.5 | 16.6 | 1.6 | 3.7 | 1.1 | 1.6 | 14.7 | 6.3 | 3.2 | 41.5 |

Table 9: elemental composition.


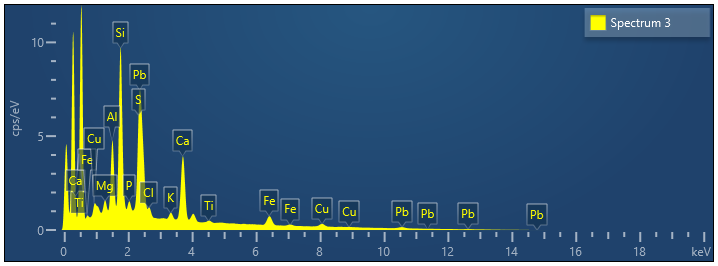


Figure 22: spectrum of spot 3.

**Sample 9 (246:10; CA220431)**


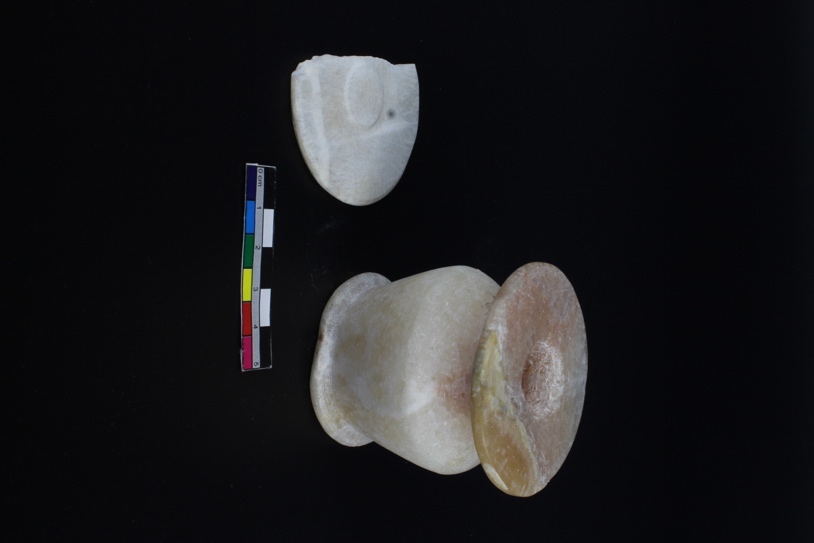


Figure 23: kohl container from Debeira East/Site 185 (246:10) (Säve-Söderbergh and Troy 1991). Photo by R. Lemos. Courtesy of Gustavianum, Uppsala University Museum.


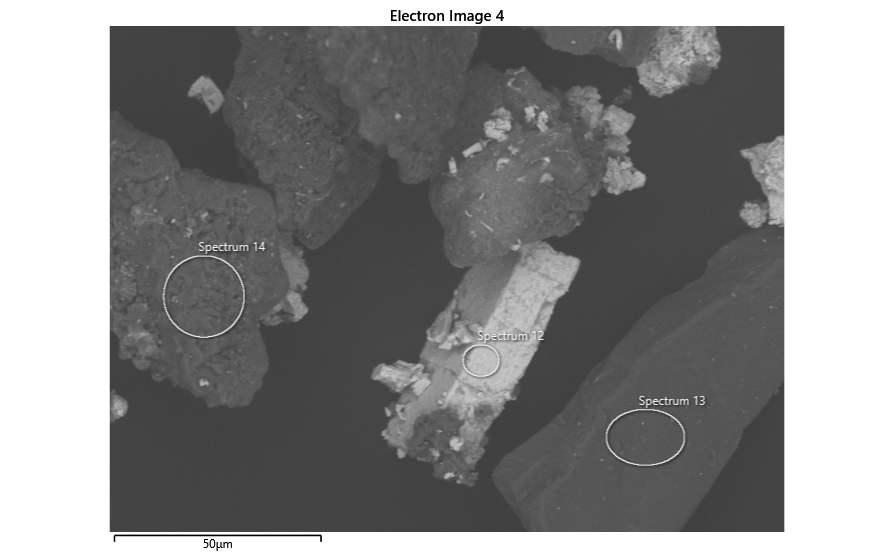


Figure 24: SEM image showing reading spots.

| Spectra | Na_2_O | MgO | Al_2_O_3_ | SiO_2_ | SO_3_ | Cl | K_2_O | CaO | FeO | SnO | TiO_2_ | PbO |
| --- | --- | --- | --- | --- | --- | --- | --- | --- | --- | --- | --- | --- |
| Spot 12 | 0.3 | 0.2 | 1.7 | 10.9 | 10.2 | / | 0.4 | 1.1 | 1.6 | 0.2 | / | 68.4 |
| Spot 13 | 0.5 | 0.4 | 2.5 | 72.1 | 3.5 | / | 0.4 | 1.4 | 2.8 | 0.1 | / | 15.6 |
| Spot 14 | 0.5 | 1.3 | 17.5 | 53.4 | 1.2 | / | 9.7 | 2.6 | 7.3 | 0.1 | 0.9 | 5.4 |

Table 10: elemental composition.


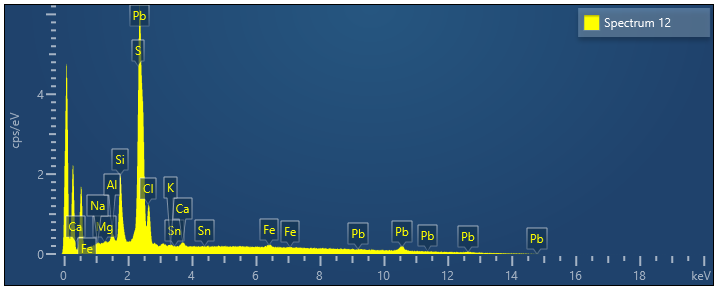


Figure 25: spectrum of spot 12.

**Sample 10 (248:7; CA220432)**


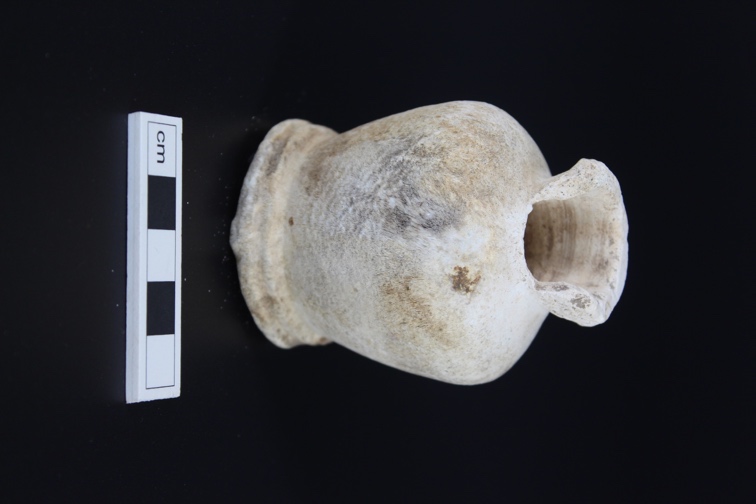


Figure 26: kohl container from Debeira East/Site 185 (248:7) (Säve-Söderbergh and Troy 1991). Photo by R. Lemos. Courtesy of Gustavianum, Uppsala University Museum.


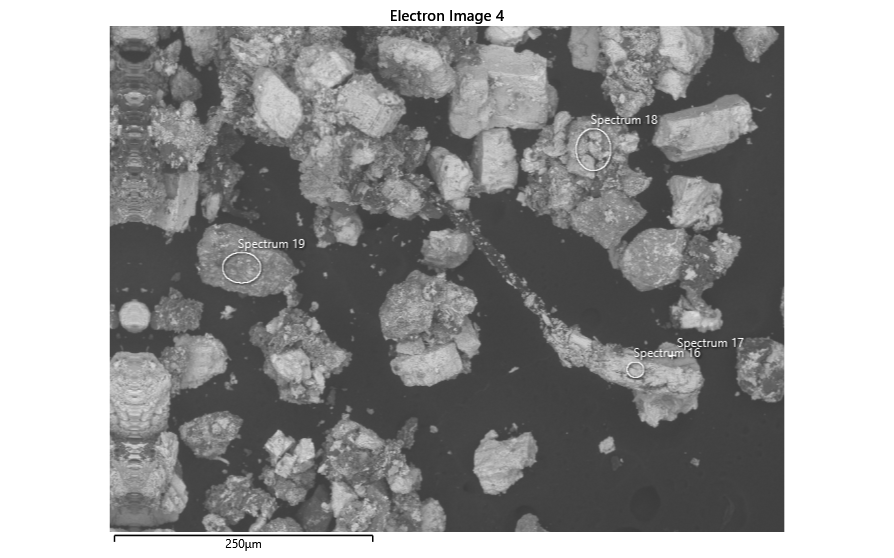


Figure 27: SEM image showing reading spots.

| Spectra | Na_2_O | MgO | Al_2_O_3_ | SiO_2_ | P_2_O_5_ | SO_3_ | CaO | TiO_2_ | FeO | ZnO | PbO |
| --- | --- | --- | --- | --- | --- | --- | --- | --- | --- | --- | --- |
| Spot 16 | / | / | 0.5 | 1.5 | / | 11.0 | 1.5 | / | 2.9 | 3.8 | 78.8 |
| Spot 17 | 1.2 | 0.3 | 0.9 | 3.6 | 0.1 | 10.6 | 3.0 | / | 14.6 | 4.9 | 60.8 |
| Spot 18 | / | 0.2 | 1.9 | 2.4 | / | 10.1 | 3.0 | / | 11.4 | 7.6 | 63.4 |
| Spot 19 | / | / | 1.1 | 1.2 | / | 5.2 | 1.0 | 0.9 | 52.4 | 4.6 | 33.6 |

Table 11: elemental composition.


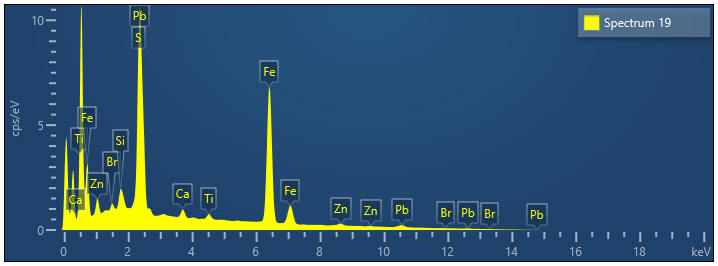


Figure 28: spectrum of spot 19.

**Sample 11 (269:1; CA220433)**


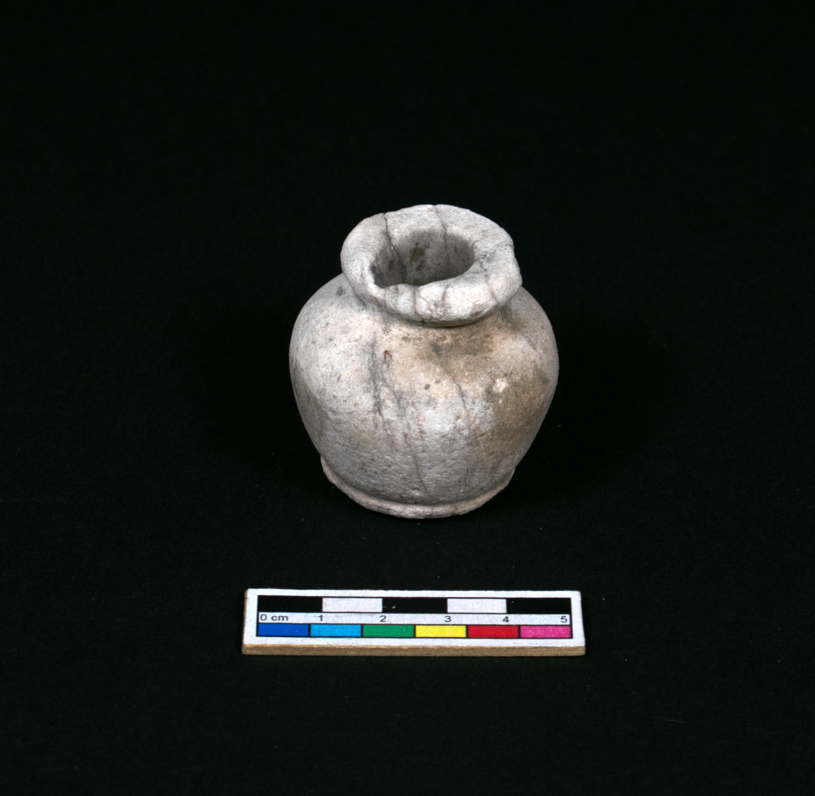


Figure 29: kohl container from Debeira East/Site 185 (269:1) (Säve-Söderbergh and Troy 1991). Photo by L. Werkström. Courtesy of Gustavianum, Uppsala University Museum.


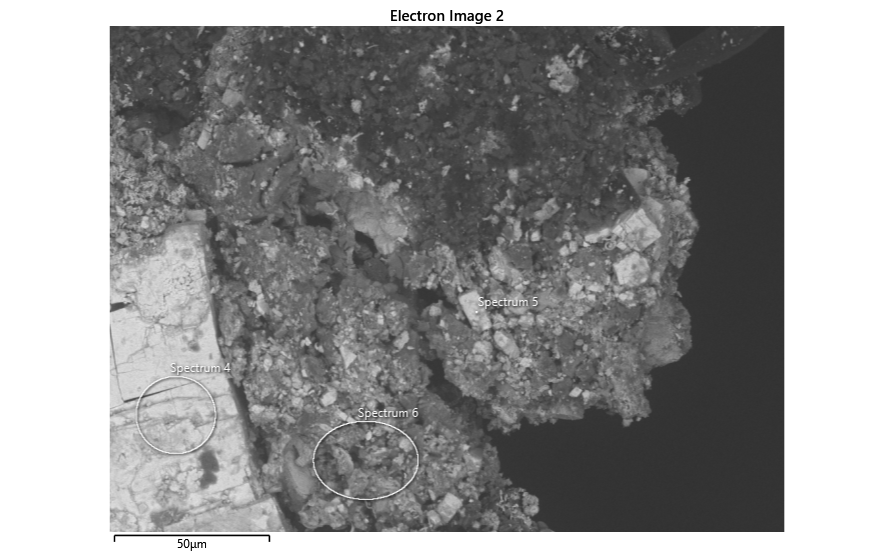


Figure 30: SEM image showing reading spots.

| Spectra | Na_2_O | MgO | Al_2_O_3_ | SiO_2_ | P_2_O_5_ | SO_3_ | CaO | FeO | ZnO | SnO | PbO |
| --- | --- | --- | --- | --- | --- | --- | --- | --- | --- | --- | --- |
| Spot 4 | 0.3 | 0.1 | 0.4 | 1.3 | / | 22.4 | 0.6 | / | / | / | 74.9 |
| Spot 5 | 0.5 | / | 0.6 | 18.5 | / | 17.2 | 0.8 | / | / | / | 62.0 |
| Spot 6 | / | / | 1.0 | 4.3 | 1.2 | 15.3 | 3.2 | 1.4 | 3.1 | / | 69.5 |

Table 12: elemental composition.


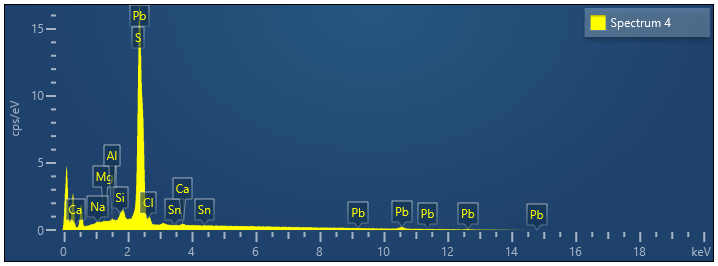


Figure 31: spectrum of spot 4.

**Sample 12 (305:1; CA220434)**


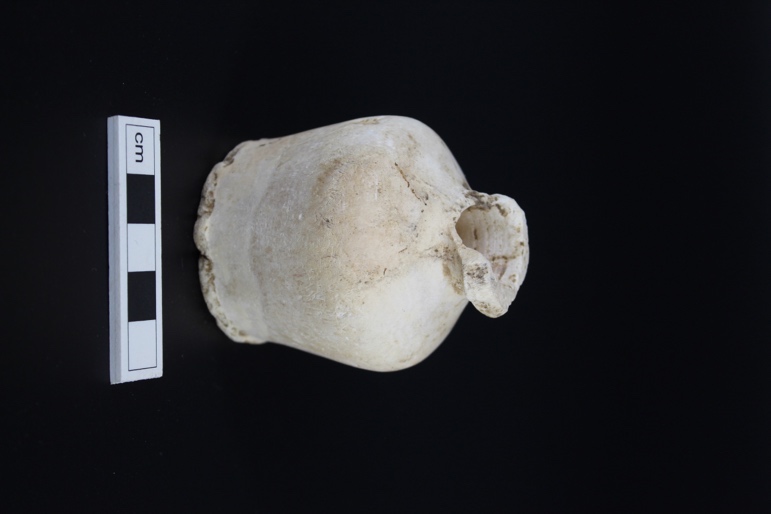


Figure 32: kohl container from Debeira East/Site 185 (305:1) (Säve-Söderbergh and Troy 1991). Photo by R. Lemos. Courtesy of Gustavianum, Uppsala University Museum.


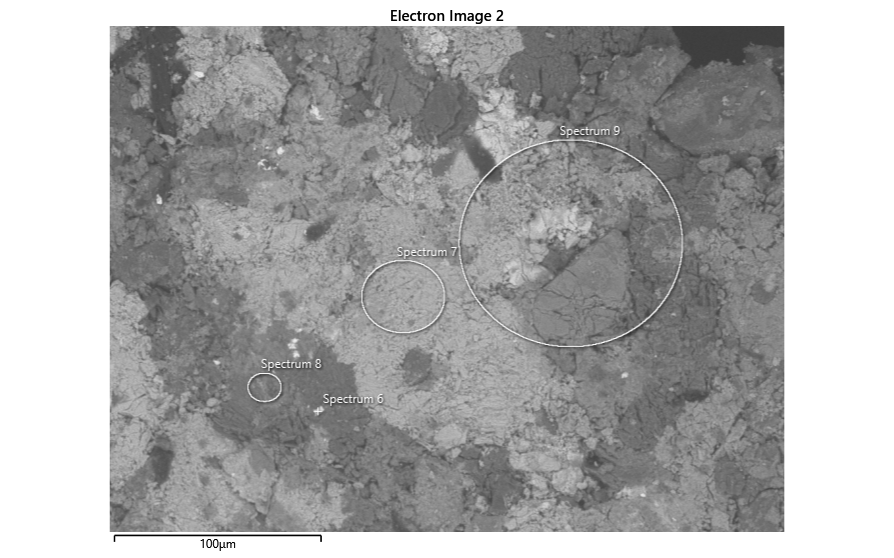


Figure 33: SEM image showing reading spots.

| Spectra | Na_2_O | MgO | Al_2_O_3_ | SiO_2_ | P_2_O_5_ | SO_3_ | K_2_O | CaO | TiO_2_ | MnO | FeO | Sb_2_O_3_ | PbO |
| --- | --- | --- | --- | --- | --- | --- | --- | --- | --- | --- | --- | --- | --- |
| Spot 6 | 0.6 | 2.6 | 14.2 | 38.3 | 1.7 | 0.8 | 1.0 | 8.4 | 0.7 | 1.4 | 6.7 | 14.8 | 7.3 |
| Spot 7 | 0.7 | 0.6 | 1.8 | 8.6 | 0.5 | 1.1 | / | 12.4 | 0.5 | / | 1.7 | 71.1 | 1.0 |
| Spot 8 | / | 2.4 | 6.4 | 46.8 | 0.9 | 0.9 | 0.8 | 9.1 | 0.7 | / | 8.1 | 23.5 | / |
| Spot 9 | 0.5 | 1.1 | 3.9 | 12.1 | 0.8 | 4.0 | / | 13.8 | 9.4 | 0.5 | 8.4 | 44.1 | 1.4 |

Table 13: elemental composition.


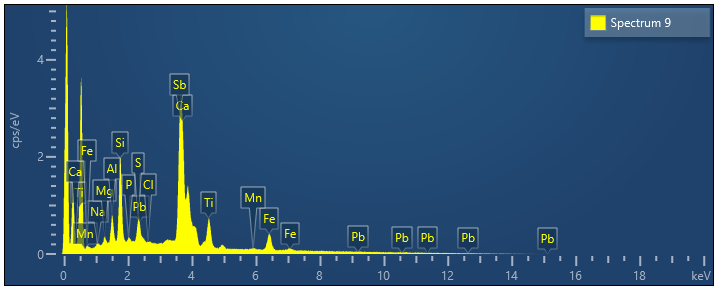


Figure 34: spectrum of spot 9.

**Sample 13 (428:1; CA220435)**


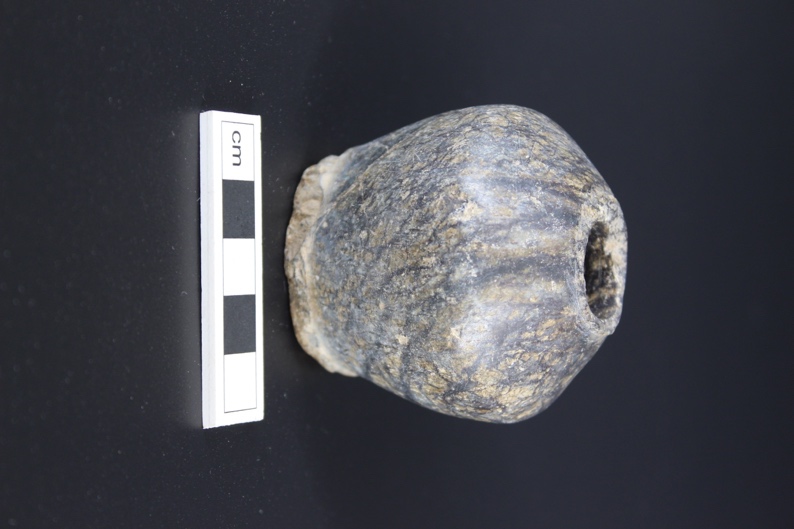


Figure 35: kohl container from Debeira East/Site 185 (428:1) (Säve-Söderbergh and Troy 1991). Photo by R. Lemos. Courtesy of Gustavianum, Uppsala University Museum.


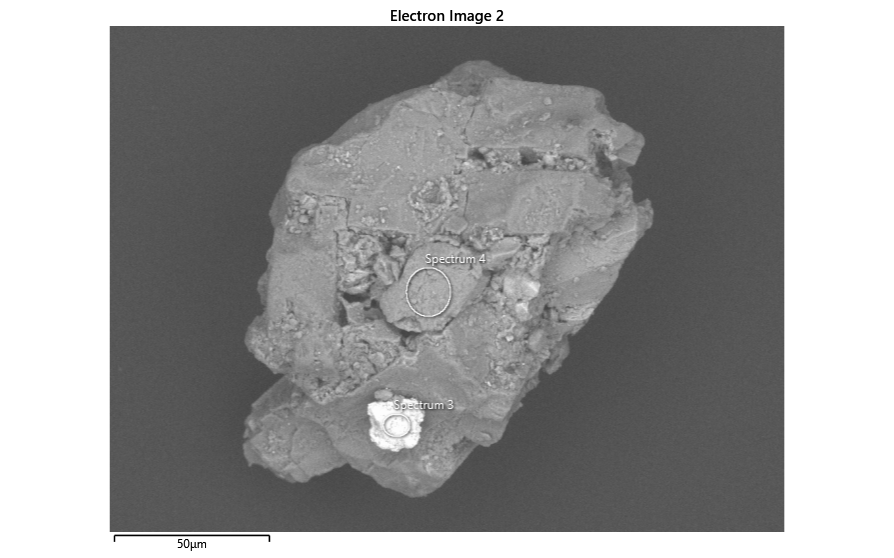


Figure 36: SEM image showing reading spots.

| Spectra | Na_2_O | MgO | Al_2_O_3_ | SiO_2_ | P_2_O_5_ | SO_3_ | K_2_O | CaO | TiO_2_ | MnO | FeO | PbO |
| --- | --- | --- | --- | --- | --- | --- | --- | --- | --- | --- | --- | --- |
| Spot 3 | 1.2 | 0.7 | 4.9 | 11.5 | 15.1 | 1.3 | 0.5 | 15.0 | 0.5 | 0.1 | 1.4 | 45.6 |
| Spot 4 | 1.2 | 8.2 | 12.3 | 53.1 | / | 0.7 | 2.4 | 1.7 | 0.5 | 0.9 | 19.1 | / |

Table 14: elemental composition.


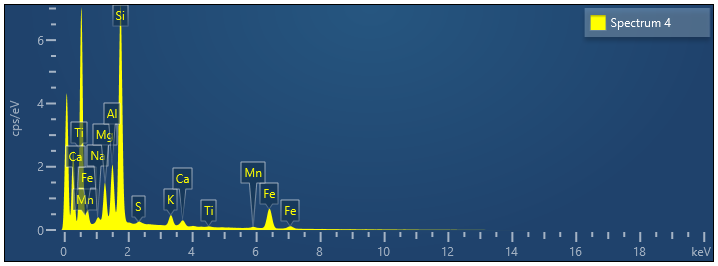


Figure 37: spectrum of spot 4.

**Sample 14 (512:20; CA220436)**


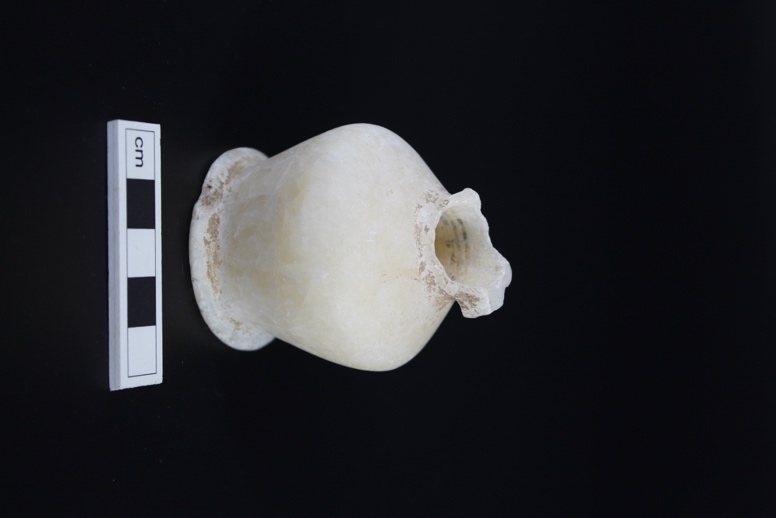


Figure 38: kohl container from Debeira East/Site 185 (512:20) (Säve-Söderbergh and Troy 1991). Photo by R. Lemos. Courtesy of Gustavianum, Uppsala University Museum.


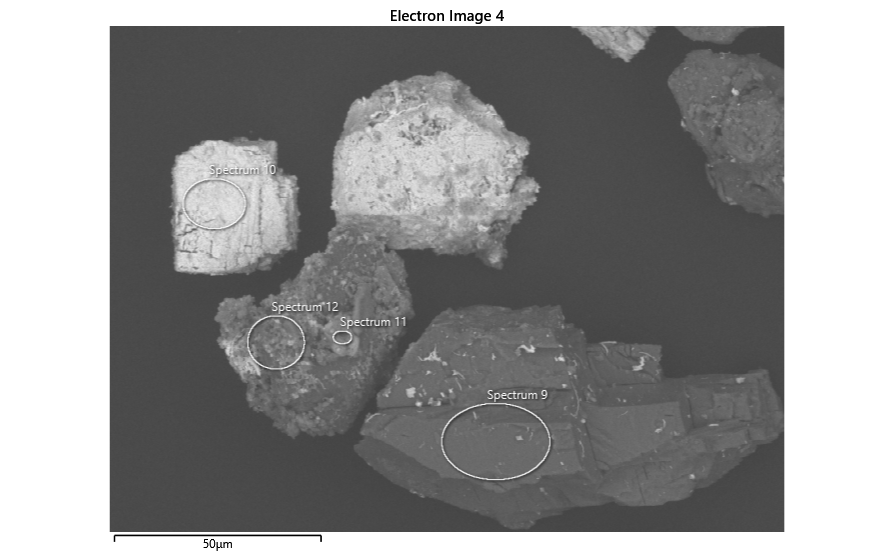


Figure 39: SEM image showing reading spots.

| Spectra | Na_2_O | MgO | Al_2_O_3_ | SiO_2_ | P_2_O_5_ | SO_3_ | K_2_O | CaO | TiO_2_ | MnO | FeO | PbO |
| --- | --- | --- | --- | --- | --- | --- | --- | --- | --- | --- | --- | --- |
| Spot 9 | 0.4 | 1.6 | 1.3 | / | / | 0.6 | / | 90.6 | / | / | 0.7 | 4.6 |
| Spot 10 | 0.3 | 0.3 | 2.2 | 4.6 | / | 5.9 | 0.2 | 1.4 | / | / | 0.9 | 78.6 |
| Spot 11 | 0.4 | 0.5 | 3.2 | 7.5 | 6.0 | 11.3 | 0.2 | 31.3 | 1.0 | 0.4 | 5.1 | 31.7 |
| Spot 12 | 0.4 | 1.3 | 19.4 | 33.5 | 1.8 | 0.4 | 0.7 | 3.8 | 2.4 | / | 6.8 | 28.1 |

Table 15: elemental composition.


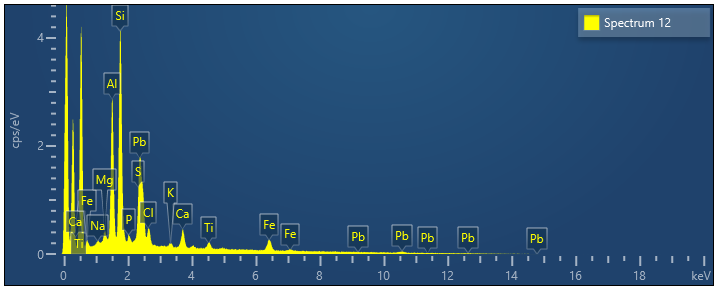


Figure 40: spectrum of spot 12.

**Sample 15 (54:1; CA220437)**


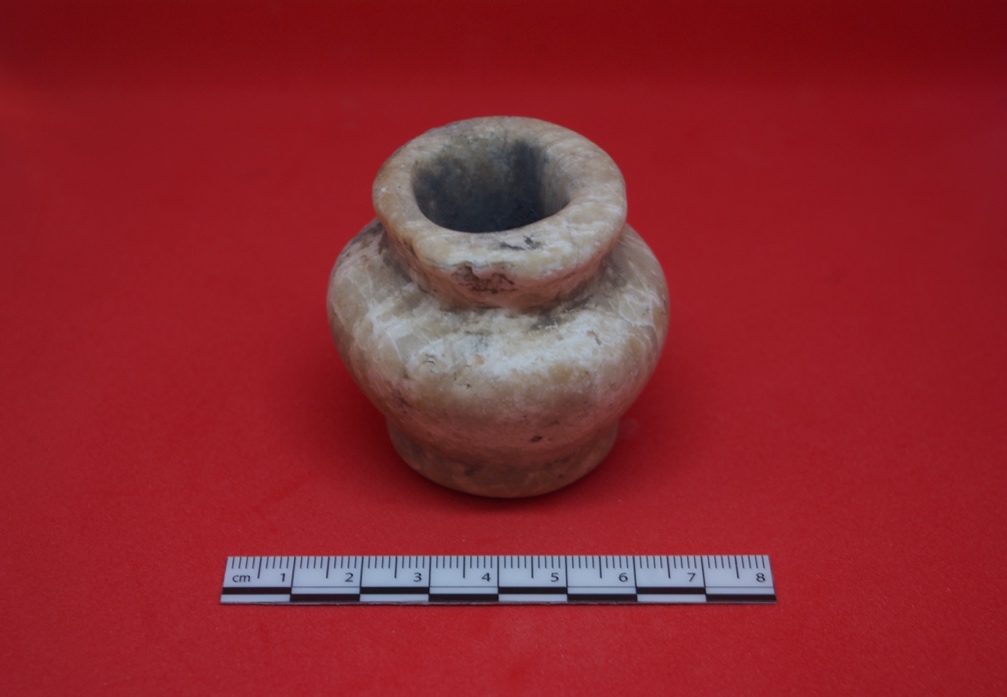


Figure 41: kohl container from Ashkeit/Site 183 (54:1) (Säve-Söderbergh and Troy 1991). Photo by R. Lemos. Courtesy of Gustavianum, Uppsala University Museum.


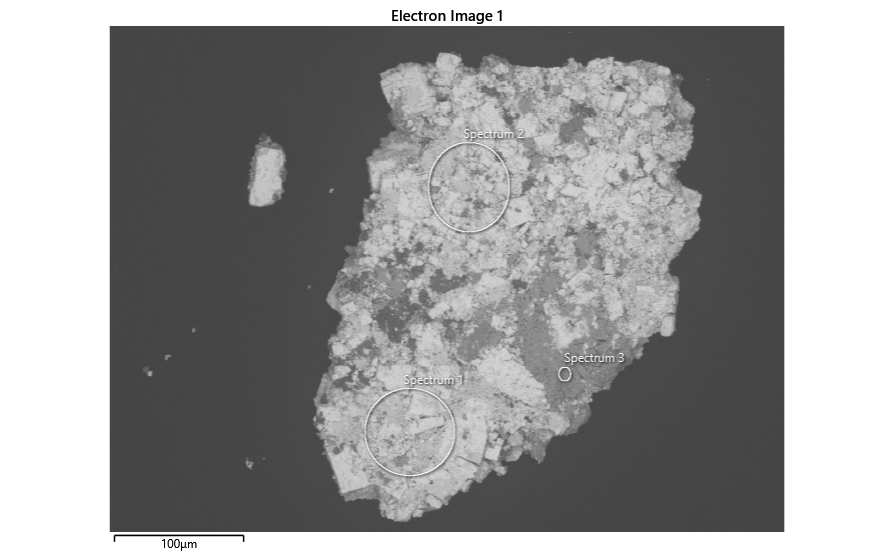


Figure 42: SEM image showing readings spots.

| Spectra | Na_2_O | MgO | Al_2_O_3_ | SiO_2_ | SO_3_ | Cl | CaO | FeO | ZnO | PbO |
| --- | --- | --- | --- | --- | --- | --- | --- | --- | --- | --- |
| Spot 1 | 0.4 | 0.2 | 0.2 | 0.5 | 15.6 | / | 0.6 | 0.2 | 5.0 | 77.4 |
| Spot 2 | 0.3 | 0.3 | 0.1 | 0.4 | 16.1 | / | 1.0 | 0.1 | 5.7 | 76.2 |
| Spot 3 | 3.3 | 0.5 | 0.1 | 0.4 | 33.6 | / | 0.1 | 0.9 | 52.2 | 8.9 |

Table 16: elemental composition.


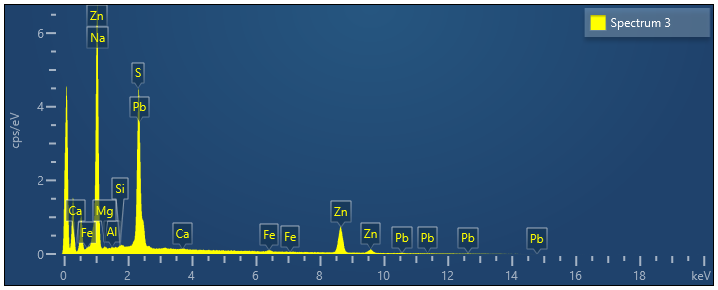


Figure 43: spectrum of spot 3.

**Sample 16 (25:3a; CA220438)**


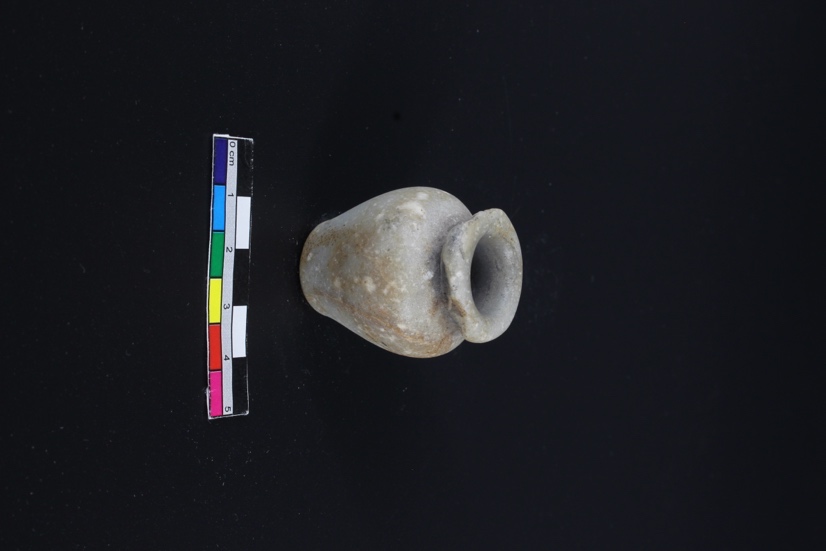


Figure 44: kohl container from Ashkeit/Site 95 (25:3a) (Säve-Söderbergh and Troy 1991). Photo by R. Lemos. Courtesy of Gustavianum, Uppsala University Museum.


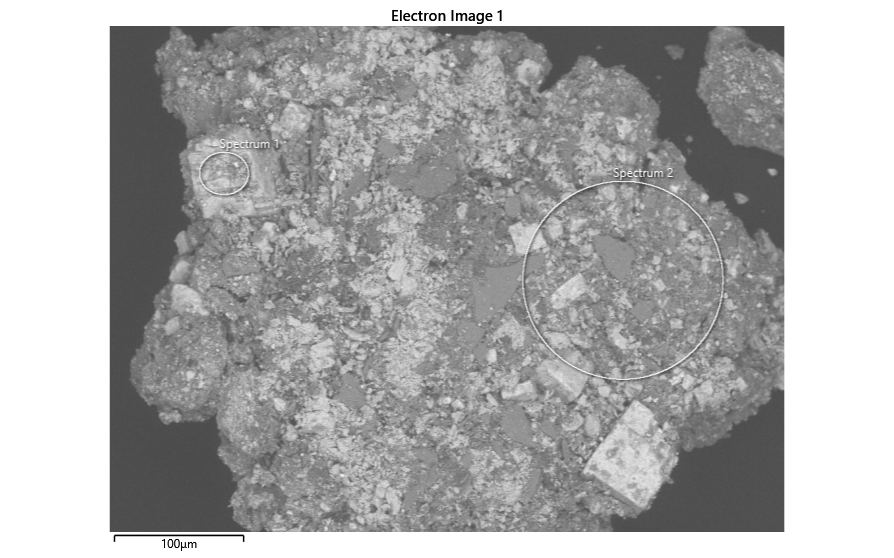


Figure 45: SEM image showing reading spots.

| Spectra | Na_2_O | MgO | Al_2_O_3_ | SiO_2_ | P_2_O_5_ | SO_3_ | K_2_O | CaO | FeO | CuO | ZnO | PbO |
| --- | --- | --- | --- | --- | --- | --- | --- | --- | --- | --- | --- | --- |
| Spot 1 | 0.7 | 0.1 | 0.3 | 1.0 | / | 20.0 | / | 1.2 | 1.7 | 1.6 | / | 72.7 |
| Spot 2 | 0.5 | 0.2 | 0.8 | 3.0 | 0.2 | 16.2 | 0.4 | 3.6 | 20.3 | / | 1.0 | 53.4 |

Table 17: elemental composition.


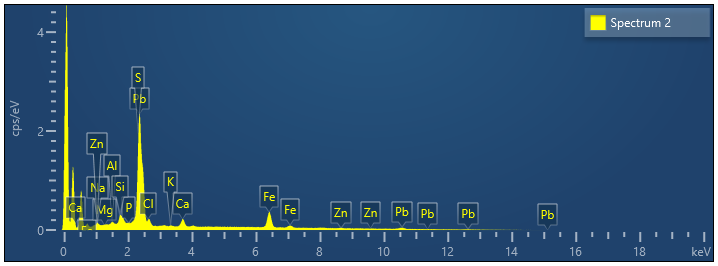


Figure 46: spectrum of spot 2.

**Sample 17 (72:1; CA220439)**


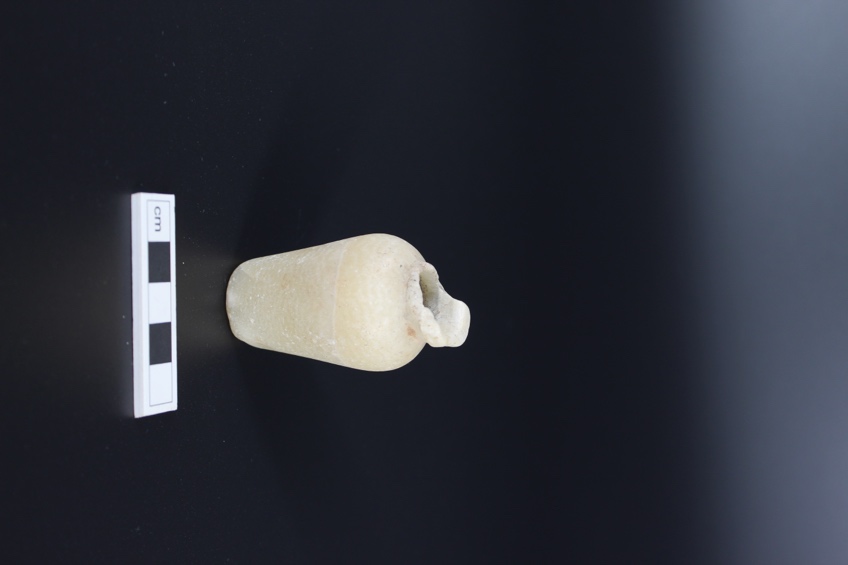


Figure 47: kohl container from Debeira East/Site 47 (72:1) (Säve-Söderbergh and Troy 1991). Photo by R. Lemos. Courtesy of Gustavianum, Uppsala University Museum.


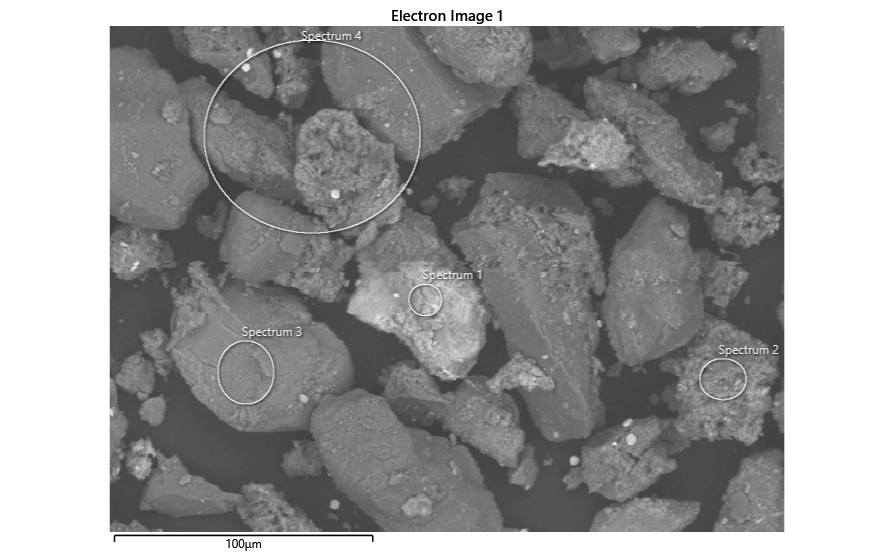


Figure 48: SEM image showing reading spots.

| Spectra | Na_2_O | MgO | Al_2_O_3_ | SiO_2_ | P_2_O_5_ | SO_3_ | K_2_O | CaO | TiO_2_ | FeO | CuO | PbO |
| --- | --- | --- | --- | --- | --- | --- | --- | --- | --- | --- | --- | --- |
| Spot 1 | 1.1 | 4.1 | 12.6 | 25.9 | 4.5 | 0.4 | 2.4 | 11.2 | 7.0 | 23.2 | / | 7.2 |
| Spot 2 | 0.9 | 5.0 | / | 35.4 | 7.5 | 1.8 | 1.5 | 29.6 | 0.4 | 4.4 | 1.1 | 5.0 |
| Spot 3 | 0.2 | 1.8 | / | 13.8 | / | 0.8 | / | 80.3 | / | 3.3 | / | / |
| Spot 4 | 0.7 | 2.5 | 11.7 | 58.6 | 2.1 | 0.4 | 1.4 | 7.2 | 1.1 | 12.3 | 0.3 | 1.8 |

Table 18: elemental composition.


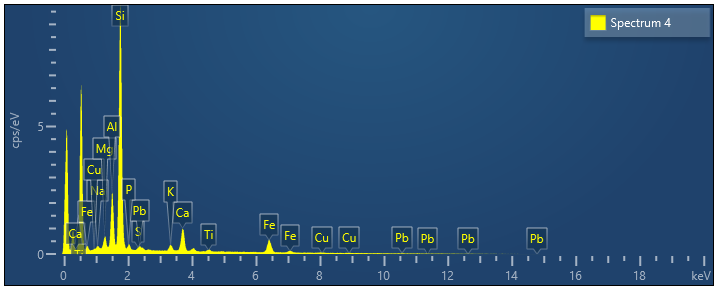


Figure 49: spectrum of spot 4.

**Sample 18 (1:5; CA220440)**


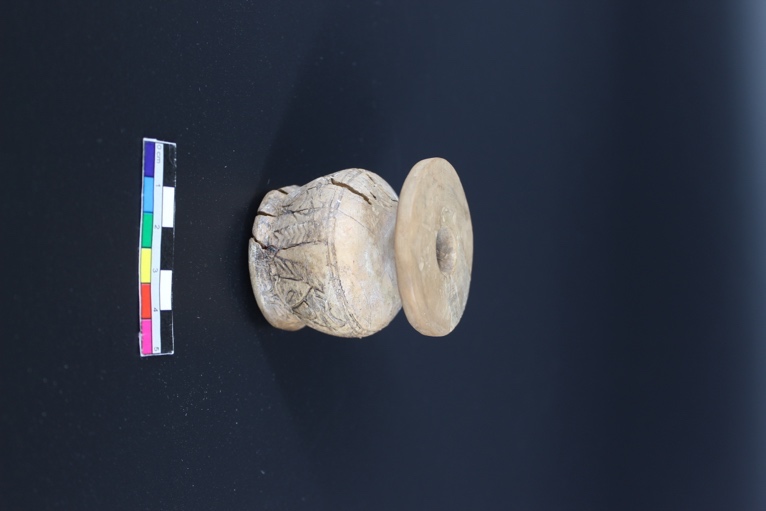


Figure 50: kohl container from Debeira East/Site 33 (1:5) (Säve-Söderbergh and Troy 1991). Photo by R. Lemos. Courtesy of Gustavianum, Uppsala University Museum.


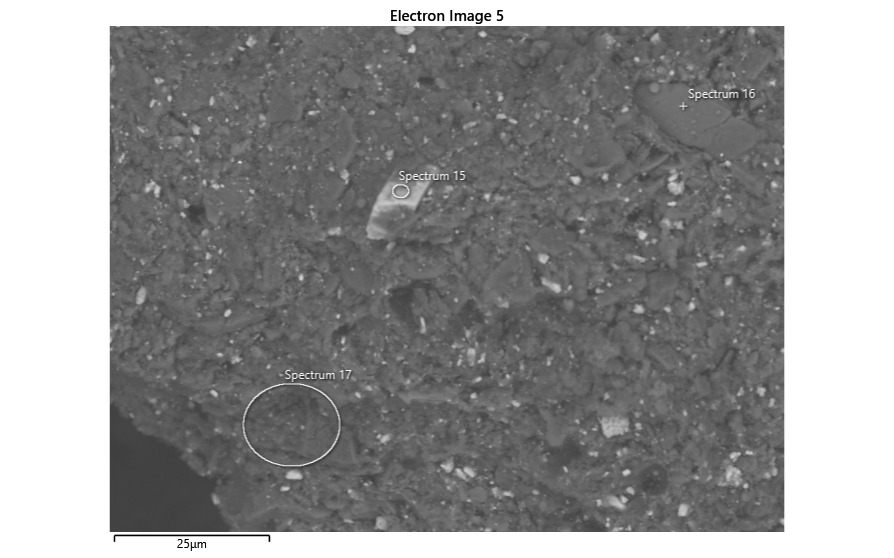


Figure 51: SEM image showing reading spots.

| Spectra | Al_2_O_3_ | SiO_2_ | SO_3_ | K_2_O | CaO | TiO_2_ | FeO | ZnO | BaO |
| --- | --- | --- | --- | --- | --- | --- | --- | --- | --- |
| Spot 15 | 7.7 | 6.4 | 16.3 | 0.3 | 1.1 | / | / | 5.4 | 62.8 |
| Spot 16 | 22.0 | 28.3 | 2.4 | 20.2 | / | 4.7 | 11.6 | 6.6 | 4.4 |
| Spot 17 | 32.4 | 40.7 | 4.0 | 1.0 | 0.7 | 6.0 | 0.6 | 11.1 | 3.4 |

Table 19: elemental composition.


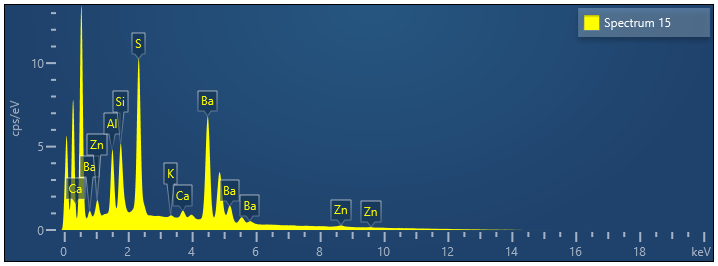


Figure 52: spectrum of spot 15.

**Sample 19 (K10-41; CA240071)**


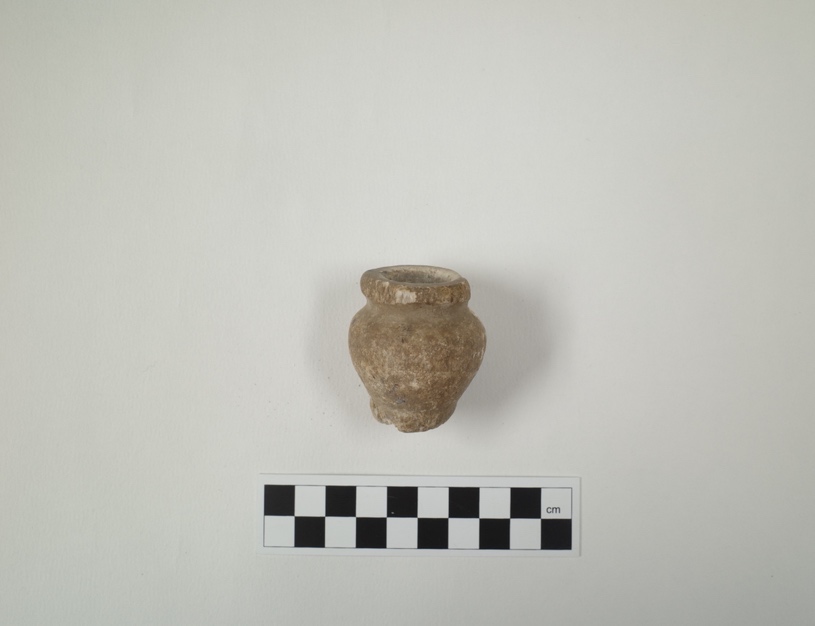


Figure 53: kohl container from Buhen (context K10-25). Photo by R. Lemos. Courtesy of the Museum of Archaeology and Anthropology, University of Cambridge.


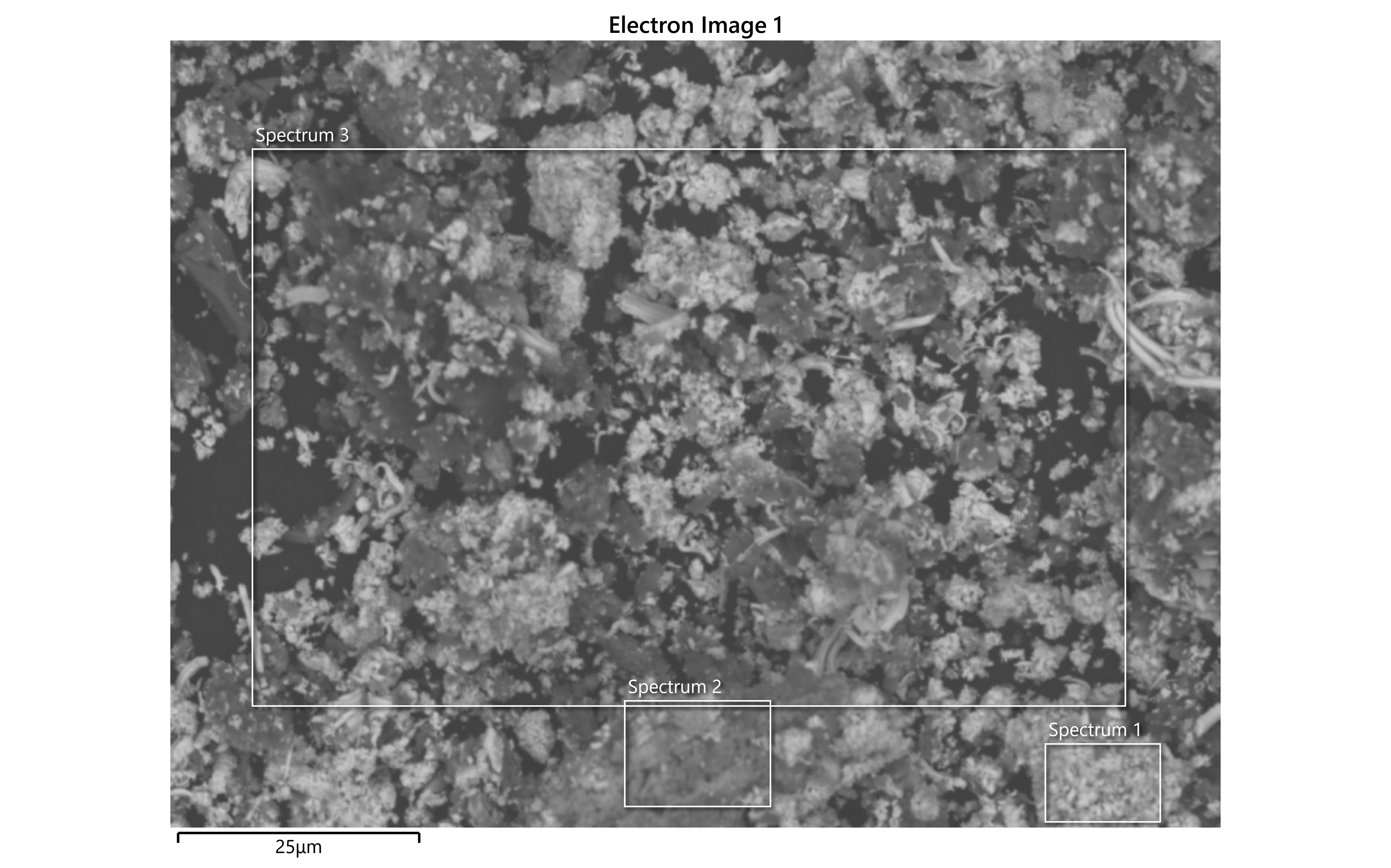


Figure 54: SEM image showing reading spots.

| Spectra | Na_2_O | MgO | Al_2_O_3_ | SiO_2_ | P_2_O_5_ | SO_3_ | K2O | CaO | TiO_2_ | FeO | MnO | ZnO | PbO |
| --- | --- | --- | --- | --- | --- | --- | --- | --- | --- | --- | --- | --- | --- |
| Spot 1 | 0.2 | 0.1 | 0.5 | 1.1 | / | 1.7 | 0.3 | 4.0 | / | 0.9 | / | / | 76.7 |
| Spot 2 | 0.9 | 1.1 | 4.9 | 16.1 | 2.0 | 7.4 | 1.6 | 9.2 | / | 4.9 | 1.4 | 1.6 | 44.5 |
| Spot 3 | 0.6 | 0.5 | 1.9 | 8.1 | 0.3 | 9.6 | 0.4 | 9.9 | 0.2 | 3.3 | / | / | 58.0 |


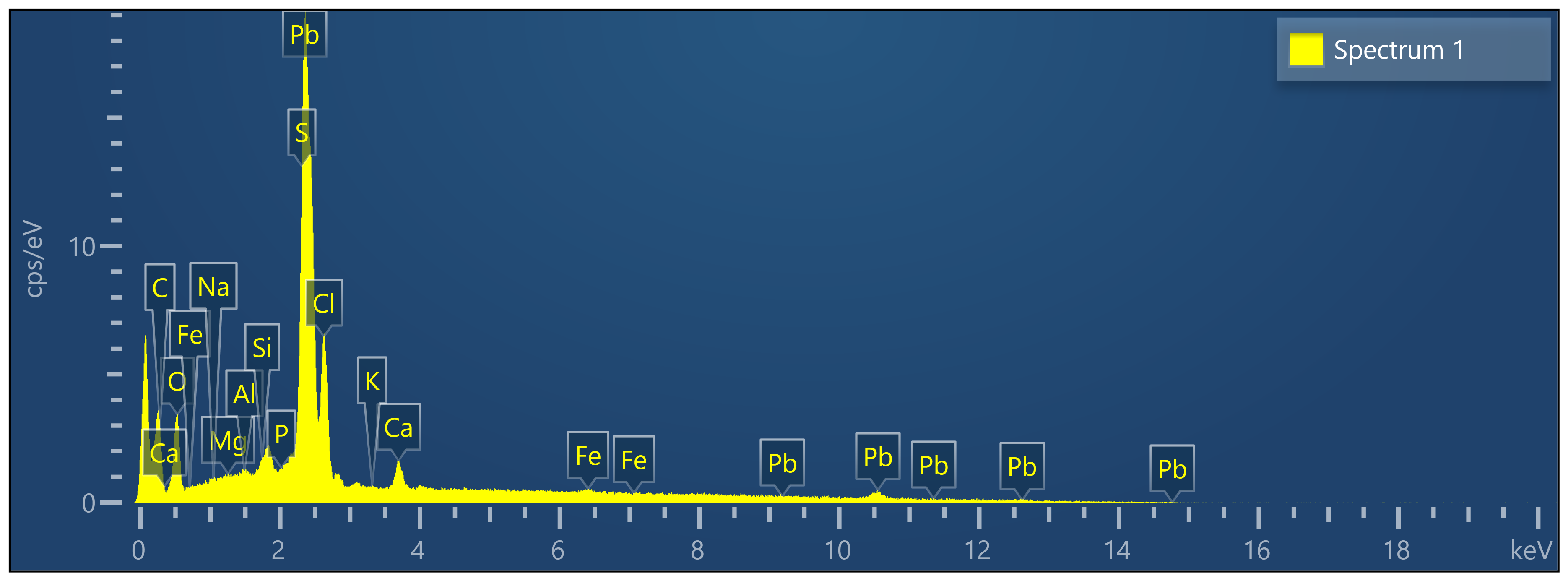


Figure 55: spectrum of spot 1.

**Sample 20 (K10-25; CA240072)**


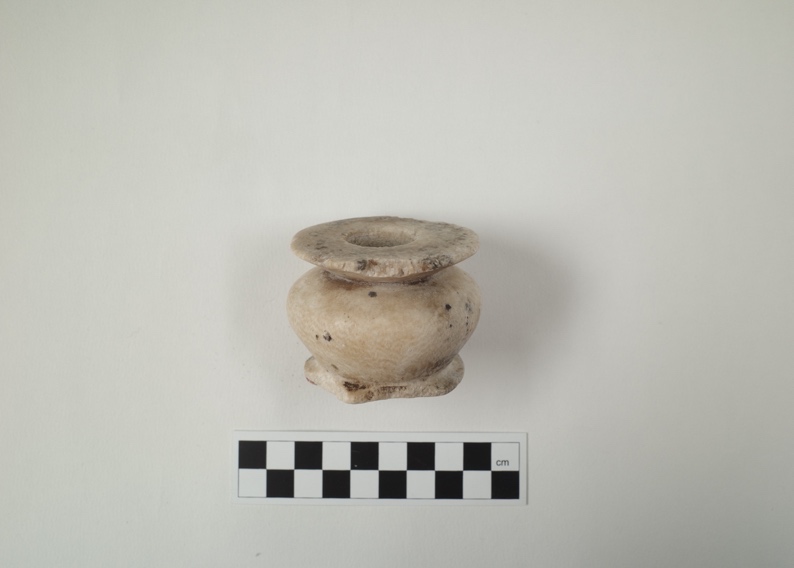


Figure 56: kohl container from Buhen (context K10-41). Photo by R. Lemos. Courtesy of the Museum of Archaeology and Anthropology, University of Cambridge.


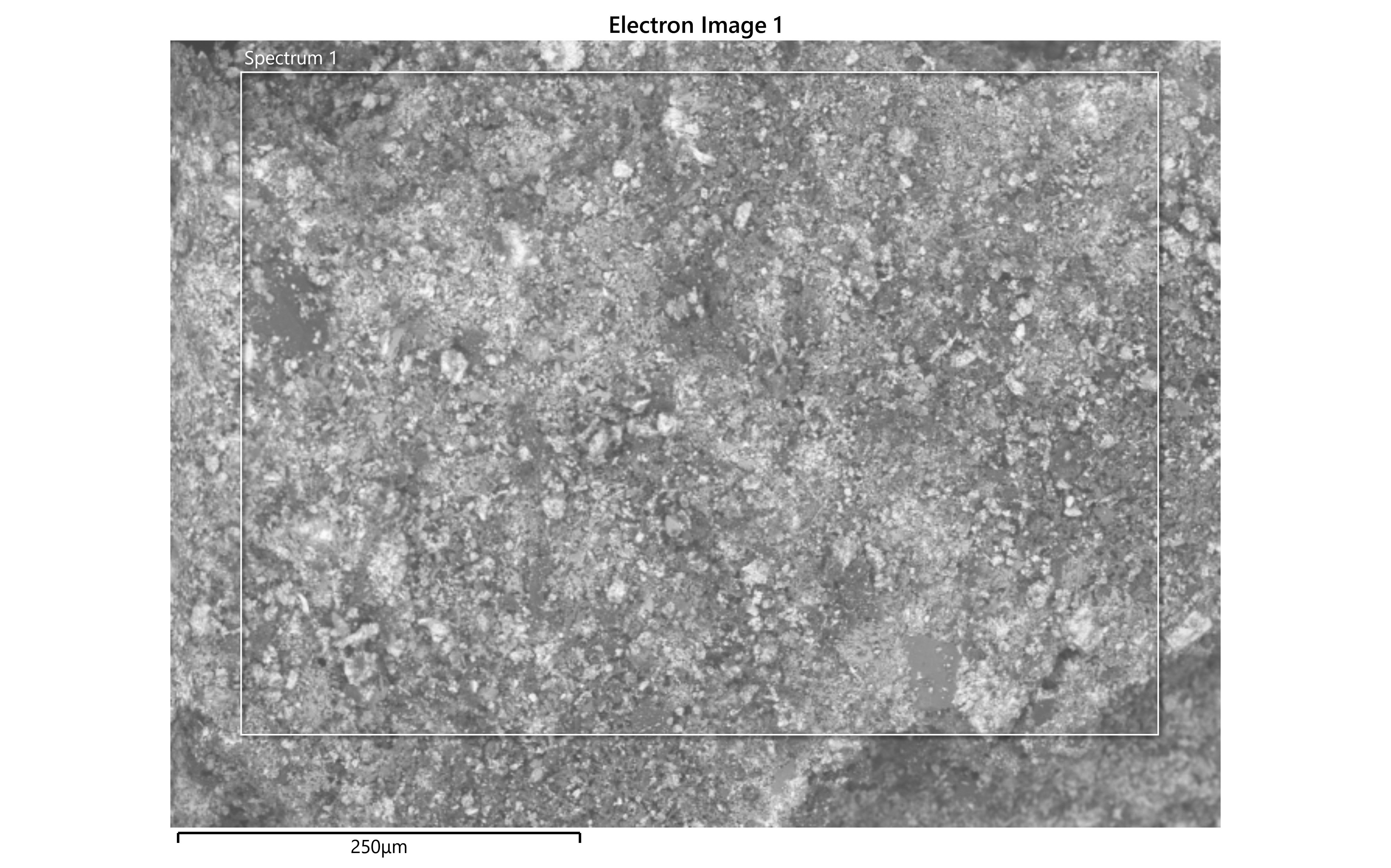


Figure 57: SEM image showing reading spots.

| Spectra | Na_2_O | MgO | Al_2_O_3_ | SiO_2_ | P_2_O_5_ | SO_3_ | K2O | CaO | TiO_2_ | FeO | MnO | ZnO | PbO |
| --- | --- | --- | --- | --- | --- | --- | --- | --- | --- | --- | --- | --- | --- |
| Spot 1 | 1.1 | 0.5 | 1.4 | 4.6 | / | 13.5 | 4.3 | 6.6 | 0.6 | 3.5 | / | 6.2 | 53.2 |


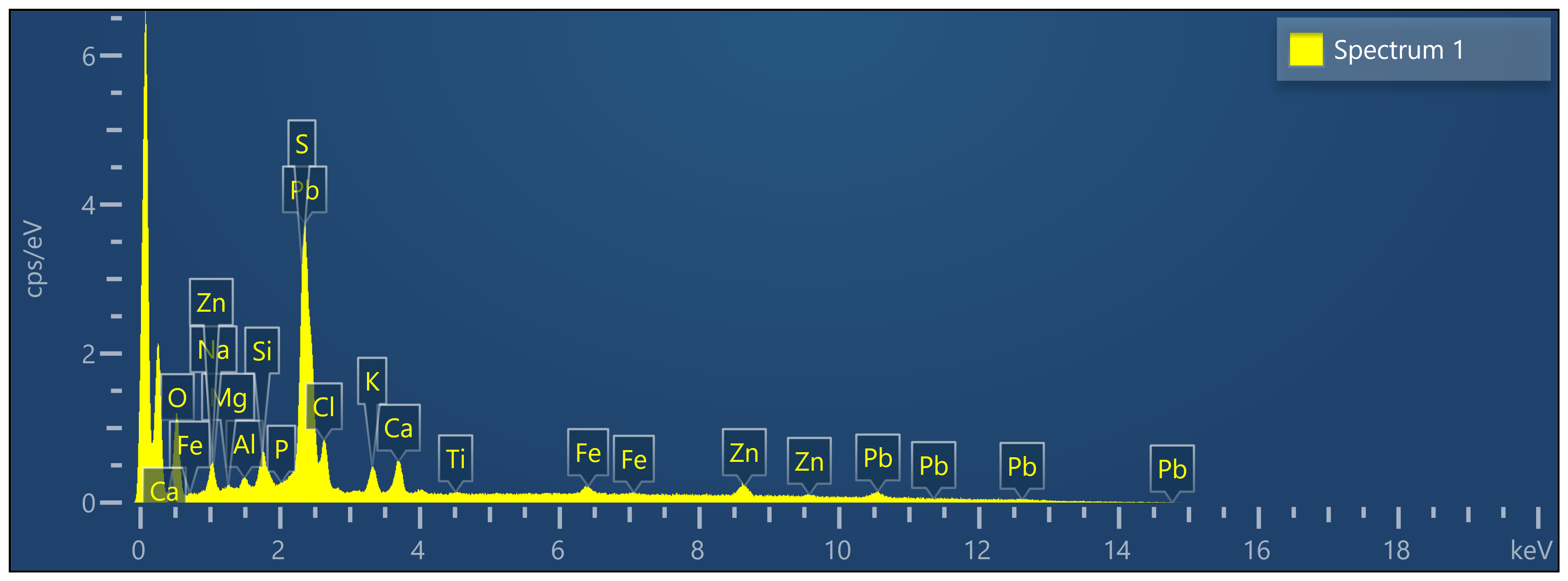


Figure 58: spectrum of reading spot 1.

**Sample 21 (442:2)^[[2]](#footnote-2)^**


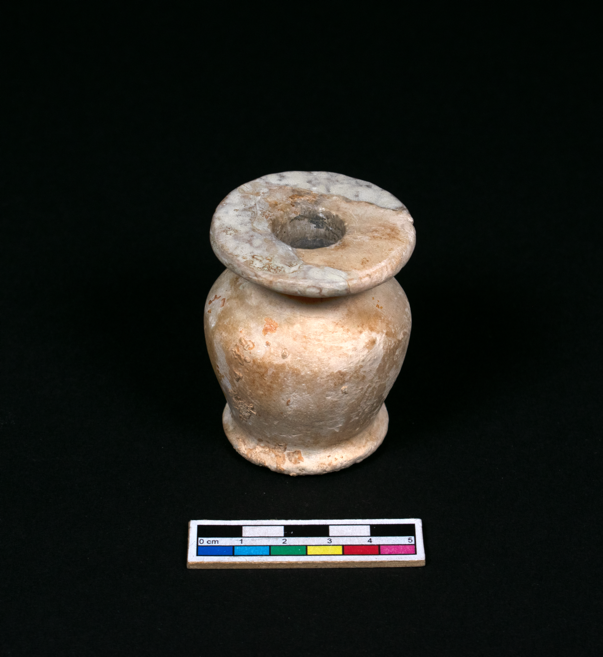


Figure 59: kohl container from Debeira East/Site 185 (442:2) (Säve-Söderbergh and Troy 1991). Photo by L. Werkström. Courtesy of Gustavianum, Uppsala University Museum.


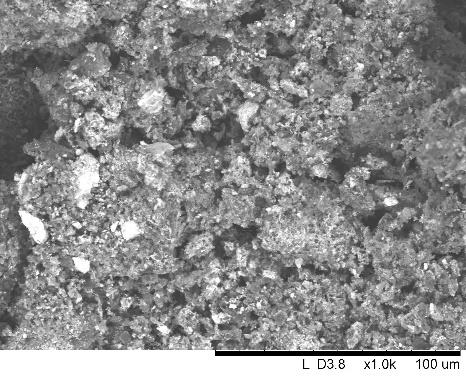


Figure 60: SEM image showing investigated area of sample.

| **Element** | **Weight %** |
| --- | --- |
| Aluminum | 3.4 |
| Silicon | 22.2 |
| Sulphur | 4.0 |
| Calcium | 7.6 |
| Lead | 62.9 |

Table 20: elemental composition.


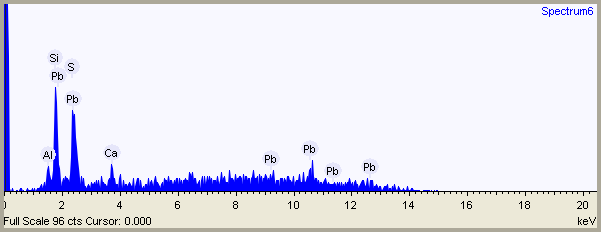


Figure 61: spectrum of area shown in figure 60.

**Sample 22^[[3]](#footnote-3)^**


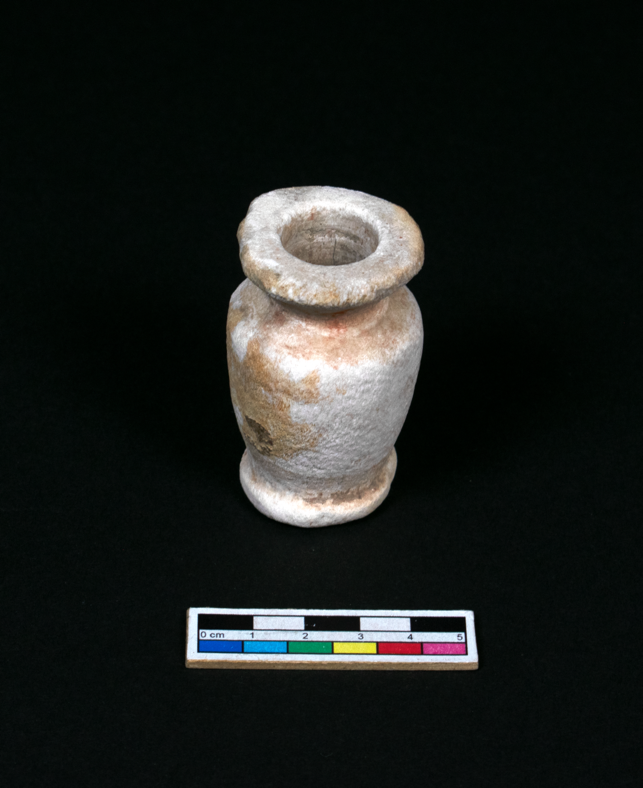


Figure 62: kohl container from Debeira East/Site 185 (30:1) (Säve-Söderbergh and Troy 1991). Photo by L. Werkström. Courtesy of Gustavianum, Uppsala University Museum.


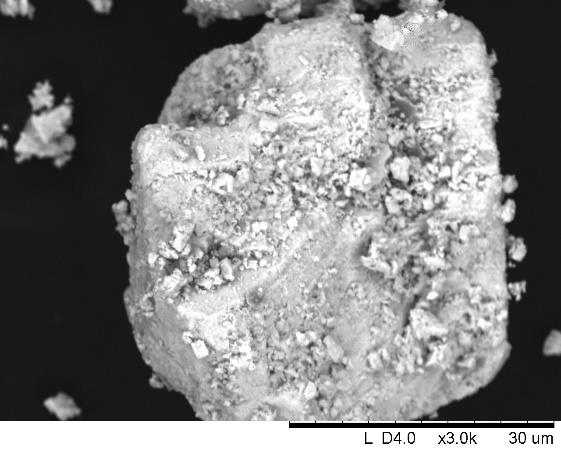


Figure 63: SEM image of investigated area of sample.

| **Element** | **Weight %** |
| --- | --- |
| Sulphur | 9.4 |
| Lead | 90.6 |

Table 21: elemental composition.


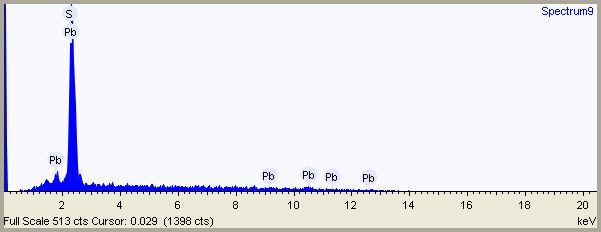


Figure 64: spectrum of area shown in figure 63.

**Sample 23^[[4]](#footnote-4)^**


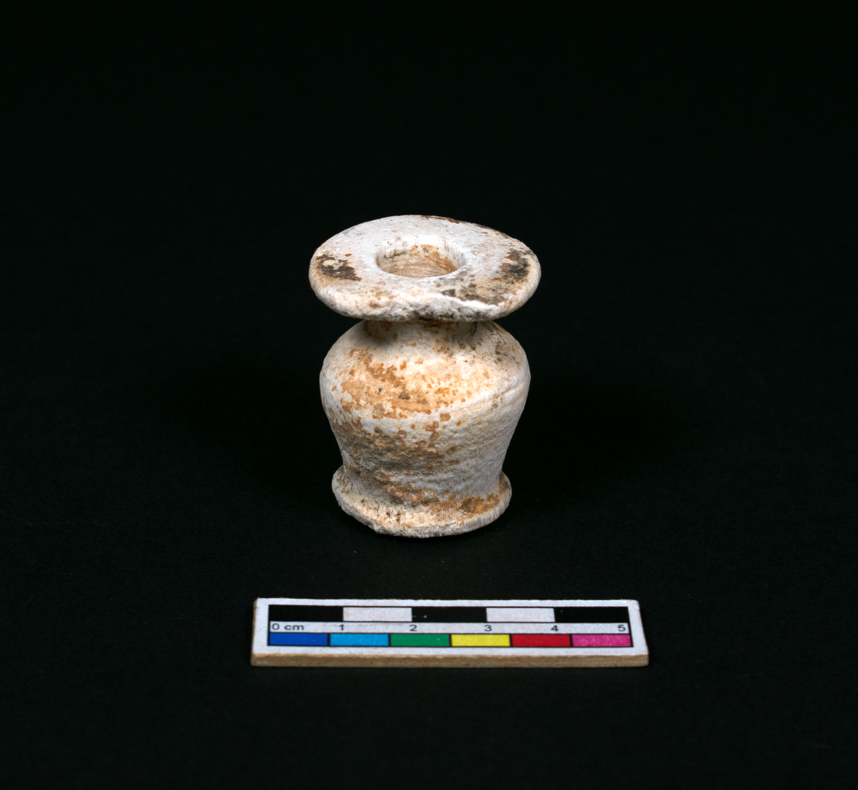


Figure 65: kohl container from Debeira East/Site 185 (274:1) (Säve-Söderbergh and Troy 1991). Photo by L. Werkström. Courtesy of Gustavianum, Uppsala University Museum.


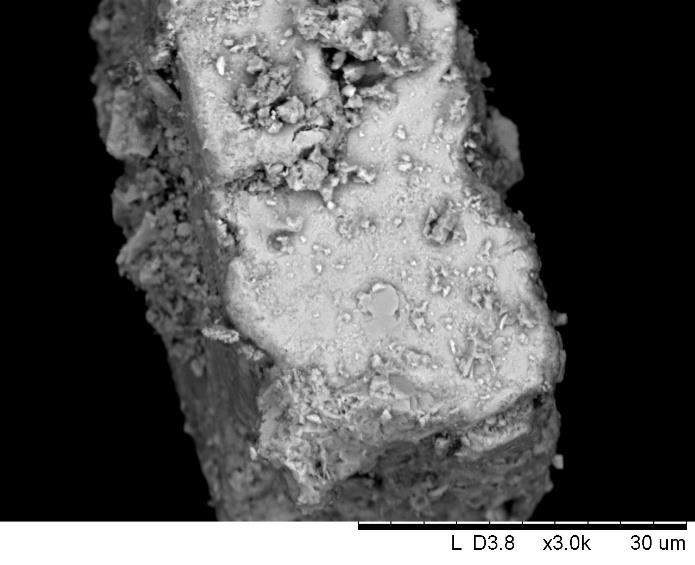


Figure 66: SEM image of investigated area of sample.

| **Element** | **Weight %** |
| --- | --- |
| Sulphur | 11.3 |
| Arsenic | N.D. |
| Lead | 88.7 |

Table 22: elemental composition.


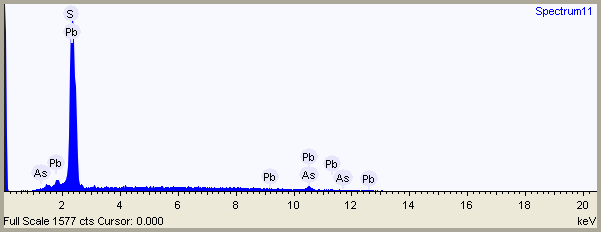


Figure 67: spectrum of area shown in figure 66.

**Sample 24^[[5]](#footnote-5)^**


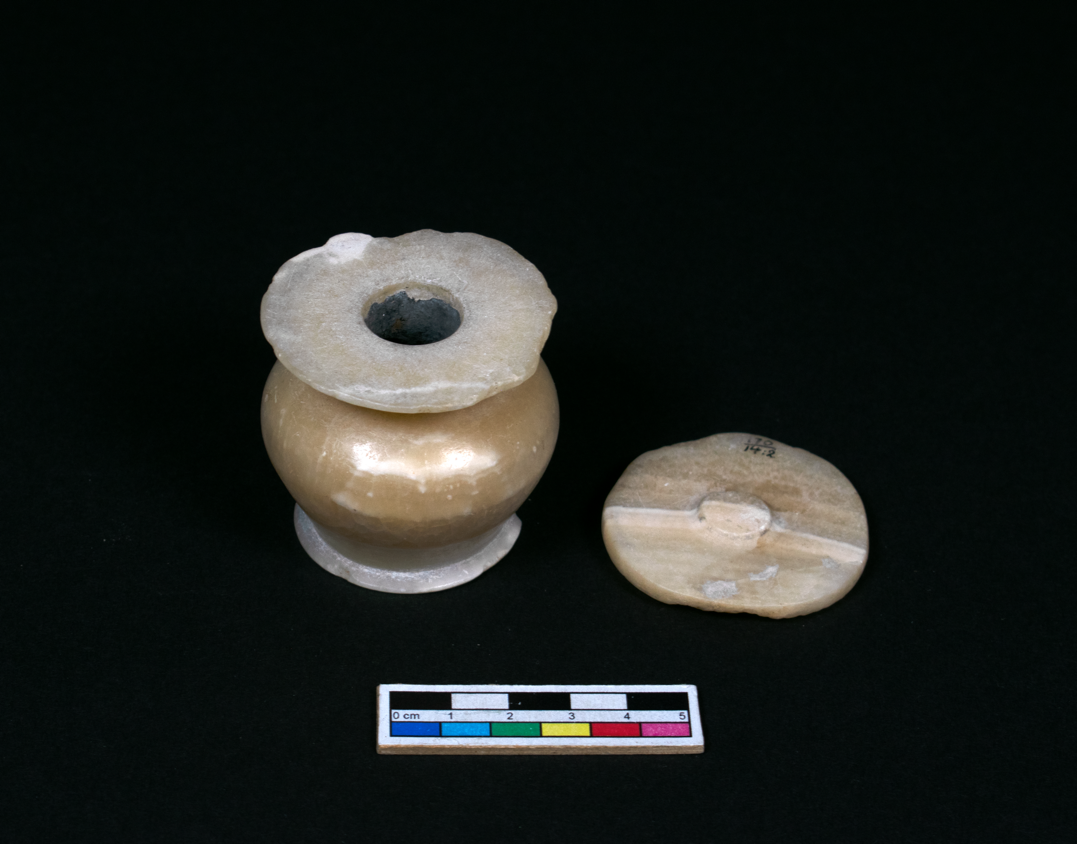


Figure 68: kohl container from Debeira East/Site 170 (14:2) (Säve-Söderbergh and Troy 1991). Photo by L. Werkström. Courtesy of Gustavianum, Uppsala University Museum.


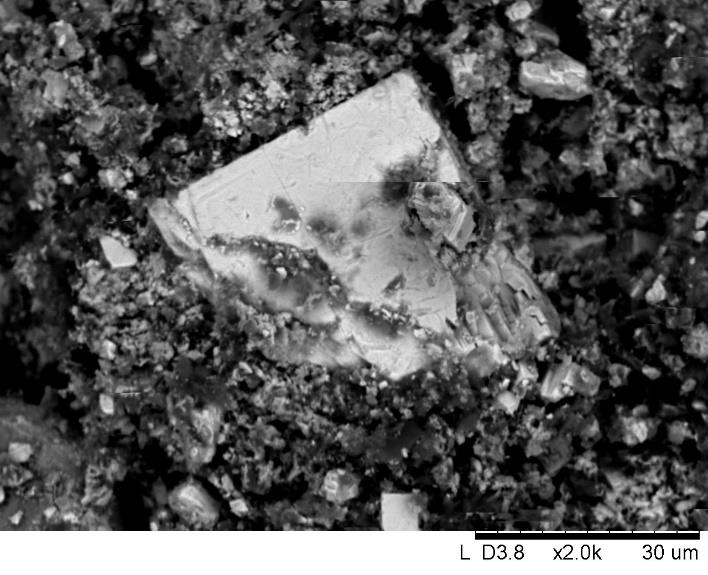


Figure 69: SEM image of investigated area of sample.

| **Element** | **Weight %** |
| --- | --- |
| Silicon | 0.8 |
| Sulphur | 12.7 |
| Calcium | 4.2 |
| Arsenic | 0.1 |
| Lead | 82.1 |

Table 23: elemental composition.


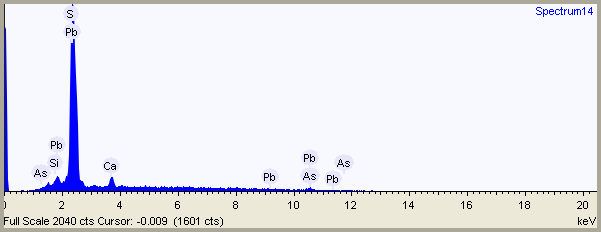


Figure 70: spectrum of area shown in figure 69.

1. CA codes given to samples analyzed at the Archaeological Science Laboratories at the University of Cambridge. [↑](#footnote-ref-1)
2. Sample analyzed at the Department of Chemistry, Uppsala University. [↑](#footnote-ref-2)
3. Sample analyzed at the Department of Chemistry, Uppsala University. [↑](#footnote-ref-3)
4. Sample analyzed at the Department of Chemistry, Uppsala University. [↑](#footnote-ref-4)
5. Sample analyzed at the Department of Chemistry, Uppsala University. [↑](#footnote-ref-5)
