# Supplementary material for: Bronze Age make-up recipes from Sudanese Lower Nubia point to a greater diversity across cultural borders in ancient Northeast Africa
Source: PLoS One. 2025 Sep 11;20(9):e0330205. doi: 10.1371/journal.pone.0330205 (PMC12425255; doi:10.1371/journal.pone.0330205)
Supplement: S2 File — (DOCX) [file pone.0330205.s002.docx]

**Supporting Information 2**

**GC-MS results**

1. Monosaccharides

| **Sample** | **Arabinose** | **Rhamnose** | **Fucose** | **Xylose** | **Mannose** | **Galactose** | **Glucose** | **Galacturonic acid** |
| --- | --- | --- | --- | --- | --- | --- | --- | --- |
| 1 |  |  |  |  |  |  |  |  |
| 2 | xx | x | x | xxx | xxx | x | xxx | x |
| 3 | xxx | x |  | x |  | xx | xxx | x |
| 4 | x | x |  | xx | xx | x | xxx | x |
| 5 | x | x |  | x | xx | x | xxx | x |
| 6 | x | x | x | xx | xx | x | xxx | x |
| 7 |  |  |  |  |  |  |  |  |
| 8 | x | x | x | xxx | xx | x | xxx | x |
| 9 | x | x |  | xx | x | x | xxx | x |
| 10 | x | x |  | xx | xx | x | xxx | x |
| 11 | x | x |  | xxx | xxx | x | xxx | x |
| 12 | x | x | x | xxx | xxx | x | xxx | x |
| 13 |  |  |  |  |  |  |  |  |
| 14 | x |  |  | xx | xx | x | xxx | x |
| 15 | xxx | x |  | xxx | xx | x | xxx | x |
| 16 | x | x | x | xx | x | xx | xxx | x |
| 17 | tr | tr |  | xx | xx | x | xxx | x |
| 18 | xx | x |  |  |  | x | xx | x |
| **Reference gums** | | | | | | | | |
| Acacia (gum Arabic) | xxx | xx |  |  |  | xx |  |  |
| Tragacanth | xx | x | x | xx |  | xx | xx | x |
| Plum | xx | x |  | x | x | xx |  |  |

1. Lipids

| **Sample** | **Lipids identified** |
| --- | --- |
| 1 | No |
| 2 | Fatty acids C8 to 18 (including odd) |
| 3 | Fatty acids C12, 14, 16, 18 |
| 4 | Fatty acids C12, 14, 16, 18 |
| 5 | No |
| 6 | No |
| 7 | No |
| 8 | No |
| 9 | No |
| 10 | No |
| 11 | No |
| 12 | No |
| 13 | No |
| 14 | No |
| 15 | Fatty acids C12, 14, 16, 18 |
| 16 | FAs 12 to 18 (including odd), diacids, monounsaturated FA C18 |
| 17 | No |
| 18 | Fatty acids 9 to 18, diacids, monounsaturated FAs 16 &18 |
