# Supplementary material for: Bronze Age make-up recipes from Sudanese Lower Nubia point to a greater diversity across cultural borders in ancient Northeast Africa
Source: PLoS One. 2025 Sep 11;20(9):e0330205. doi: 10.1371/journal.pone.0330205 (PMC12425255; doi:10.1371/journal.pone.0330205)
Supplement: S4 File — (DOCX) [file pone.0330205.s004.docx]

**تشير وصفات مكياج العصر البرونزي من شمال السودان إلى تنوع أكبر عبر الحدود الثقافية في شمال شرق إفريقيا القديمة**

**الملخص**

كشفت الدراسات العلمية السابقة للكحل وغيره من المستحضرات التجميلية من مصر القديمة عن تنوع كبير في المواد والوصفات المستخدمة في مناطق وفترات زمنية مختلفة. ومع ذلك، لم تُدرج عينات من النوبة السودانية في الدراسات العلمية لمواد التجميل المستخدمة على طول وادي النيل. للمرة الأولى، تم تحليل 24 عينة من الكحل ومستحضرات التجميل الأخرى من النوبة السفلى السودانية في العصر البرونزي (حوالي 2055-1070 قبل الميلاد) باستخدام المجهر الضوئي، الفصل اللوني للغاز/قياس الطيف الكتلي، المجهر الإلكتروني الماسح مع مطياف للأشعة السينية، مطيافية الأشعة تحت الحمراء باستخدام تحويل فورييه، وتحليل حيود الأشعة السينية. توسع هذه الدراسة معرفتنا باستخدام مستحضرات التجميل في وادي النيل القديم من خلال إدراج عينات من السودان، كما تعمق فهمنا لمواد التجميل في شمال شرق أفريقيا القديم عبر دراسة عينات من سياقات أثرية محددة بدقة.

يسلط النهج التحليلي المتعدد المستخدم هنا الضوء على تنوع الوصفات المستخدمة من قبل المجتمعات المختلفة في وادي النيل الأوسط خلال العصر البرونزي. يهيمن وجود كبريتيدات الرصاص على معظم العينات، ولكنها تظهر في خلطات متنوعة مع الكوارتز، والطين، والكالسيت، والجبس، ومركبات الزنك، بالإضافة إلى صمغ النباتات والدهون الحيوانية. علاوة على ذلك، تظهر النتائج ولأول مرة استخدام كربونات الكالسيوم الاصطناعية في الخلطات التجميلية.

بجانب توسيع معرفتنا بتركيبات مستحضرات التجميل في شمال شرق إفريقيا القديمة، تشير دراستنا إلى أن التنوع الملحوظ عبر الحدود الثقافية بين مصر والنوبة في عصر البرونز يعكس مفاهيم متميزة للجسد والمظهر.

**الكلمات المفتاحية:** مستحضرات التجميل؛ الكحل؛ النوبة؛ السودان؛ مصر؛ العصر البرونزي؛ المجهر الضوئي؛GC-MS ؛ SEM-EDS؛ FTIR؛ XRD.
